# Mex-3 RNA binding family member A (MEX3A)/circMPP6 complex promotes colorectal cancer progression by inhibiting autophagy

**DOI:** 10.1038/s41392-024-01787-3

**Published:** 2024-04-02

**Authors:** Ri-Xin Chen, Shui-Dan Xu, Min-Hua Deng, Shi-Hui Hao, Jie-Wei Chen, Xiao-Dan Ma, Wei-Tao Zhuang, Jing-Hua Cao, Yong-Rui Lv, Jin-Long Lin, Si-Yu Li, Gui-Bin Qiao, Dan Xie, Feng-Wei Wang

**Affiliations:** 1 Department of Thoracic Surgery, Guangdong Provincial People’s Hospital (Guangdong Academy of Medical Sciences), Southern Medical University, Guangzhou, 510080 China; 2 State Key Laboratory of Oncology in South China, Guangdong Provincial Clinical Research Center for Cancer, Sun Yat-sen University Cancer Center, Guangzhou, 510060 China; 3 Medical Research Institute, Guangdong Provincial People’s Hospital (Guangdong Academy of Medical Sciences), Southern Medical University, Guangzhou, 510080 China; 4 Department of Pathology, Sun Yat-sen University Cancer Center, Guangzhou, 510060 China

**Keywords:** Tumour biomarkers, Non-coding RNAs

## Abstract

RNA-binding proteins (RBPs)-RNA networks have contributed to cancer development. Circular RNAs (circRNAs) are considered as protein recruiters; nevertheless, the patterns of circRNA-protein interactions in colorectal cancer (CRC) are still lacking. Processing bodies (PBs) formed through liquid-liquid phase separation (LLPS) are membrane-less organelles (MLOs) consisting of RBPs and RNA. Previous evidence suggests a connection between PBs dynamics and cancer progression. Despite the increasingly acknowledged crucial role of RBPs and RNA in the accumulation and maintenance of MLOs, there remains a lack of specific research on the interactions between PBs-related RBPs and circRNAs in CRC. Herein, we identify that MEX-3 RNA binding family member A (MEX3A), frequently upregulated in CRC tissues, predicts poorer patient survival. Elevated MEX3A accelerates malignance and inhibits autophagy of CRC cells. Importantly, MEX3A undergoes intrinsically disordered regions (IDRs)-dependent LLPS in the cytoplasm. Specifically, circMPP6 acts as a scaffold to facilitate the interaction between MEX3A and PBs proteins. The MEX3A/circMPP6 complex modulates PBs dynamic and promotes UPF-mediated phosphodiesterase 5A (*PDE5A*) mRNA degradation, consequently leading to the aggressive properties of CRC cells. Clinically, CRC patients exhibiting high MEX3A expression and low PDE5A expression have the poorest overall survival. Our findings reveal a collaboration between MEX3A and circMPP6 in the regulation of mRNA decay through triggering the PBs aggregation, which provides prognostic markers and/or therapeutic targets for CRC.

## Introduction

Colorectal cancer (CRC) ranks third in incidence and second in cancer-related mortality worldwide,^1^ according to the global cancer statistics in 2020. Despite advancements in available surgical and chemotherapeutic treatment, the prognosis associated with CRC patients remains dismal due to the challenges of recurrence and distant organ metastasis. There is a great urgent need for novel therapeutic agents to treat CRC more effectively. Hence, the identification of key molecules and the elucidation of their underlying mechanisms involved in the disease progression of CRC represents an important area of cancer research.

Liquid–liquid phase separation (LLPS) is a dynamic and intricate physiological process in which biomolecules undergo a transition from a homogeneous environment to distinct sparse and dense phases, contributing to cellular quality control and biochemical network organization.^2^ It is widely acknowledged that LLPS, driven by the cumulative interactions of proteins or RNAs, condenses various biomacromolecules into liquid-like membrane-less aggregate compartments, including nucleoli, paraspeckles, Cajal bodies, and promyelocytic leukemia (PML) bodies in the nucleus, as well as stress granules (SGs) and processing bodies (PBs) in the cytoplasm.^3^ PBs are known to form through the co-assembly of RNAs with RNA binding proteins (RBPs), notably with mRNA degradation machinery components or ribonucleoproteins (RNPs), involving in mRNA decay and translational repression.^4^ Recent investigations have begun to unveil evidence linking LLPS to tumor initiation and progression, deepening our comprehension of the underlying pathophysiological mechanisms and providing potential avenues for therapeutic targeting.^2^ Interestingly, PBs homeostasis is closely related to various stresses such as hypoxia, nutrient starvation et al. Given the essential role of stressful conditions in the tumor microenvironment, the dysregulation of PBs formation might play a role in cancer pathological process. Nevertheless, the involvement of PBs mediators in cancer malignant behavior has rarely been studied.

MEX-3 RNA binding family member A (MEX3A), is a subtype of RBPs that functions in various RNA metabolism-related processes including mRNA stability,^5,6^ and miRNA transport between the nucleus and cytoplasm.^7^ Accumulating evidence has suggested the involvement of MEX3A in cancer progression such as lung cancer,^6^ glioma,^8^ and breast cancer.^9^ Multiple studies have demonstrated that MEX3A takes part in intestinal differentiation and cell stemness.^10^ MEX3A labels the slowly cycling subpopulation of Lgr5+ intestinal stem cells. MEX3A-high cells in mice intestines exhibit resistance to the deleterious effects of chemotherapy or irradiation and play an important role in the regeneration of impaired crypts.^11^ Furthermore, MEX3A-high cells in CRC patient-derived organoids behave as a bona fide drug-tolerant persister cell population, thus contributing to the modulation of relapse following chemotherapy.^12^ Li et al. reported a regulatory mechanism in which miR‐6887‐3p binds to the 3’ UTR of *MEX3A* and thus inhibits MEX3A/RAP1GAP/MAPK signaling pathway to enhance CRC oncogenesis.^5^ Yang et al. identified MEX3A functions as an oncoprotein in CRC, elucidating the E2F3/MEX3A/KLF4 axis as a crucial coordinator of cancer stem cell self-renewal and differentiation.^13^ Current research reveals that activation of IGF-1R initiates MEX3A-mediated degradation of RIG-I, resulting in the inhibition of IFN-I-related immune cells in the CRC tumor microenvironment and thereby facilitating CRC advancement.^14^ These findings shed light on the intricate regulatory networks mediated by MEX3A in intestinal development and CRC progression. However, as an RBP, the exact MEX3A-RNAs network in CRC remains largely unclear. Circular RNAs (circRNAs), a new type of noncoding RNAs (ncRNAs), are acknowledged to be dysregulated in many diseases. In cancer biology, circRNAs exert well-characterized function as tumor suppressors or oncogenic drivers through a series of mechanisms. Similar to other ncRNAs, circRNAs mainly exert function by sponging microRNA^15^ or interacting with RBP.^16^ Additionally, circRNAs may proceed to be translated as templates.^17^ Accumulating evidence has revealed that circRNAs could serve as protein decoys, scaffolds, and recruiters, affecting the interaction or localization of proteins.^18–20^ A previous study demonstrated that circVAMP3 drives LLPS of CAPRIN1 to promote SG formation and inhibit c-Myc translation, thereby inhibiting hepatocellular carcinoma malignancy.^21^ However, the precise patterns of circRNA-protein interactions in the assembly of membrane-less granules remain poorly understood and require further investigation. Hence, elucidating the molecular partners, including RBPs, through which circRNAs affect PBs dynamics in cancer cell biology, will be essential for developing effective interventions.

In the present study, we characterize the contributing role of MEX3A in CRC cell malignant properties and autophagy activity. Our findings reveal that MEX3A undergoes LLPS and coordinates the recruitment of PBs components in living cells. Notably, circMPP6 emerges as a pivotal scaffold, fostering the interaction between MEX3A and PBs components, thereby facilitating PBs formation in CRC cells. The MEX3A/circMPP6 complex exerts a substantial influence on the degradation of phosphodiesterase 5A (*PDE5A*) mRNA, thereby eliciting CRC cell aggressiveness. Clinically, CRC patients with elevated expression of MEX3A and attenuated expression of PDE5A exhibit a worse outcome. Collectively, our comprehensive insights shed light on the important role of the RBP/circRNA complex in modulating PBs homeostasis, providing optimistic prospects for prognostic assessment and/or therapeutic interventions for CRC patients.

## Results

### MEX3A is highly expressed in CRCs and predicts the poor prognosis

**Fig. 1:**
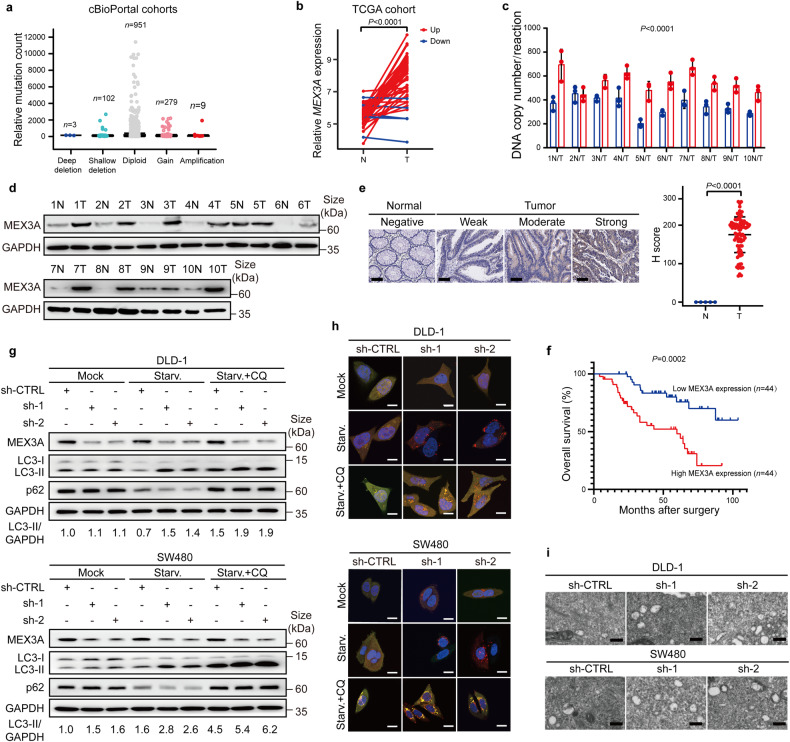
MEX3A is highly expressed in CRCs and suppresses autophagy of CRC cells. **a** Copy number variations (CNVs) of the MEX3A gene in 1344 patients from the 19 CRC cohorts in online cBioPortal tool (http://www.cbioportal.org). **b** mRNA expression of *MEX3A* in 50 tumors (T) and paired adjacent normal tissues (N) in the TCGA CRC cohort. **c** Absolute quantification of copy numbers of MEX3A in 10 CRC tissues (T) and matched adjacent noncancerous tissues (N) from the SYSUCC. Y-axes showing the DNA molecules per PCR reaction. **d** Western blotting of MEX3A protein expression in 10 CRC tissues (T) and matched adjacent noncancerous tissues (N) from the SYSUCC. GAPDH was used as the loading control. **e** Left, representative IHC staining for MEX3A in normal samples (left) and CRCs (right). Scale bar, 100 μm. Right, quantified H-score of cytosolic MEX3A in CRC tissues (T) and noncancerous tissues (N). **f** Kaplan-Meier analysis of overall survival (OS) in CRC patients with low versus high MEX3A expression from SYSUCC cohorts (*n* = 88). **g** Western blotting for LC3 conversion and p62 expression in control or MEX3A-knockdown cells in the absence or presence of serum or Chloroquine (CQ). GAPDH was used as an internal control. **h** Detection of autophagic flux with the mRFP-GFP-LC3 reporter in indicated cells. Yellow puncta represent autophagosomes (mRFP+/GFP+), and red puncta represent autolysosomes (mRFP+/GFP−). Scale bar, 10 μm. **i** Representative transmission electron microscope (TEM) images of the autophagosomes and/or autolysosomes in indicated cells. Scale bar, 500 nm. Data are represented as mean ± S.D. (**c**, **e**), and the *P* value was determined by two-tailed paired Student’s *t* test (**b**, **c**) or two-tailed unpaired Student’s *t* test (**e**) or Log-rank test (**f**)

The previous study has uncovered the landscape of genomic alterations of RBPs by cross-cancer analysis.^22^ By utilizing online cBioPortal tool (http://www.cbioportal.org) including 19 CRC datasets, we noted that cancer-related RBP MEX3A was distinctly and significantly amplified and overexpressed in human CRCs (Fig. 1a, b). We then examined the copy number and protein expression of MEX3A in CRCs. Consistently, MEX3A tended to be markedly amplified and upregulated in CRCs compared with matched adjacent noncancerous tissues (Fig. 1c, d). Next, we performed immunohistochemistry (IHC) staining of 88 CRC tissues and 5 matched noncancerous colorectal tissues obtained from Sun Yat-sen University Cancer Center (SYSUCC). The results unveiled a remarkable elevation of cytoplasmic MEX3A expression in CRC tissues compared with adjacent normal tissues (Fig. 1e). High expression of MEX3A in CRCs was positively associated with clinical stage and advanced T and N stages (*P* < 0.05; Supplementary Table S1, 2), and predicted poorer patient overall survival (OS) (*P* < 0.05; Fig. 1f). These data suggest that the upregulation of MEX3A exerts an oncogenic effect in CRC pathogenesis.

### MEX3A accelerates the malignant properties and suppresses autophagy of CRC cells

To investigate the role of MEX3A in CRC cells, we constructed two stable MEX3A-knockdown CRC cell lines (DLD-1 and SW480) using two short hairpin RNAs (shRNAs), and one stable MEX3A-overexpression cell line HCT116 (Supplementary Fig. S1a). MEX3A knockdown significantly attenuated CRC cells colony formation and migration in vitro (Supplementary Fig. S1b, c), whereas MEX3A overexpression enhanced the malignant properties (Supplementary Fig. S1d, e). In the subcutaneous xenograft model, MEX3A knockdown substantially reduced the in vivo tumorigenicity and tumor weights (Supplementary Fig. S1f). Conversely, nude mice injected with MEX3A-overexpressed cells had a significantly increased tumor growth (Supplementary Fig. S1g). Overall, MEX3A aggravates the CRC progression.

Given that RBPs mainly exert their distinct role by interaction with mRNAs, we performed RNA-binding protein immunoprecipitation and deep sequencing (RIP-Seq) to detect MEX3A-binding mRNAs. Kyoto Encyclopedia of Genes and Genomes (KEGG) pathway analysis illustrated that mRNAs bound to MEX3A were chiefly enriched in the FoxO signaling pathway, which was known as a vital regulatory circuit of autophagy activity,^23^ and the autophagy pathway (Supplementary Fig. S2a). These results indicate that autophagy may play an essential role in MEX3A-mediated CRC progression.

Then we assessed whether MEX3A participates in autophagy occurrence. Upon serum starvation, knockdown of MEX3A promoted the autophagic flux as indicated by an increase of LC3-II, a hallmark of autophagic activity. This effect was prolonged by treatment with the lysosomotropic alkalizing agent Chloroquine (CQ). As an indicator of autophagy flux inhibition, p62 is known to degrade in autolysosomes.^24^ Starvation-stimulated p62 decay occurred in control cells, while MEX3A knockdown reduced p62 accumulation, suggesting the increased autophagy flux. Interestingly, starvation-induced reduction in p62 levels in both control and MEX3A-knockdown cells was blocked by CQ treatment (Fig. 1g). In contrast, MEX3A overexpression decreased LC3-II accumulation and increased p62 levels in starved cells (Supplementary Fig. S2b). We further performed the mRFP-GFP-LC3 assay to monitor the autophagy flux. While the LC3B signal was diffuse in the cytoplasm under basal conditions, starvation triggered the formation of fluorescent LC3 puncta. Notably, MEX3A-knockdown cells had more autophagosomes and/or autolysosomes, as suggested by an increase of yellow and/or red puncta (Fig. 1h, Supplementary Fig. S2c). Conversely, MEX3A overexpression potently impeded the autophagosome maturation (Supplementary Fig. S2d). Ultra-structurally, the number of autophagosomes and autolysosomes was strongly increased in CRC cells upon MEX3A knockdown by transmission electron microscope (TEM) (Fig. 1i). In contrast, MEX3A-overexpressed CRC cells greatly inhibited the formation of autophagosomes and autolysosomes (Supplementary Fig. S2e). Collectively, these data illustrate that MEX3A inhibits the autophagy program and thus promotes CRC progression.

### MEX3A undergoes IDRs-dependent LLPS in vitro and in vivo

**Fig. 2:**
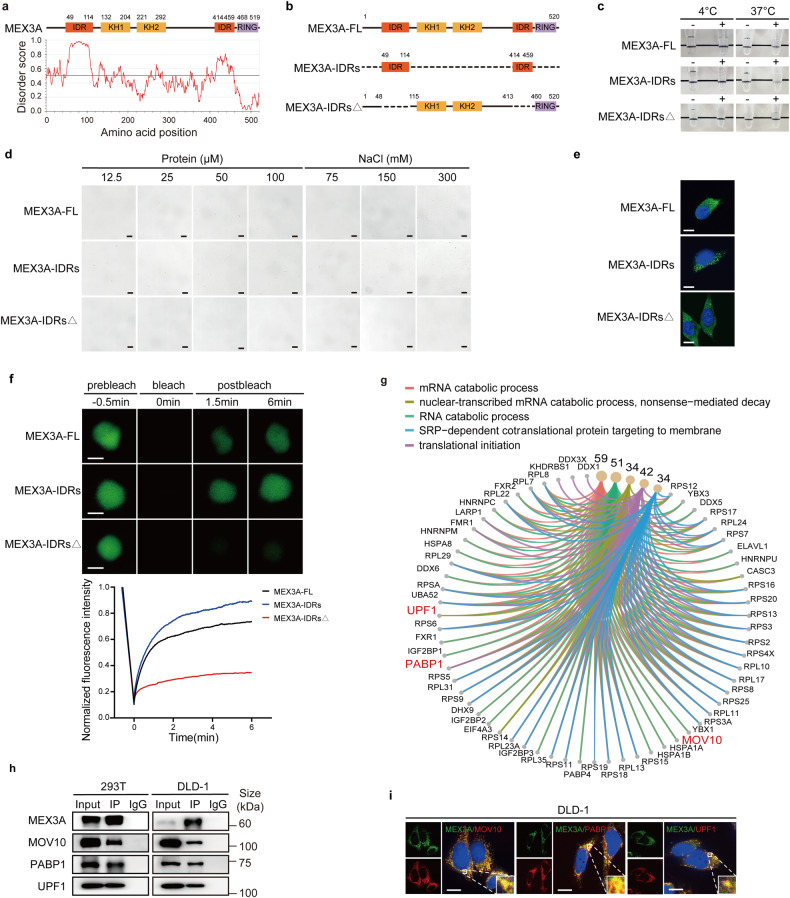
MEX3A undergoes IDRs-dependent LLPS and physically interacts with PBs components. **a** Domain structure and the intrinsically disordered regions (IDRs) of MEX3A. The IUPred score greater than 0.5 indicates disordered, as determined by the online IUPred2A algorithm (http://iupred2a.elte.hu). **b** The schematic diagram representing the MEX3A full-length and IDRs truncated mutant plasmids. **c** Representative images of turbidity associated with droplet formation. Tubes contain either only buffer (−) or recombination proteins (+) at the indicated temperature. **d** Representative phase-contrast microscopy images of MEX3A-FL and MEX3A-IDRs droplets formed in buffers containing 150 mM NaCl and different concentrations of protein (left) or 100 μM protein and different concentrations of NaCl (right). Scale bar, 50 μm. **e** Immunofluorescence (IF) assay showing EGFP-MEX3A-FL, EGFP-MEX3A-IDRs, EGFP-MEX3A-IDRs△(green) formed cytoplasmic puncta in DLD-1 cells. Nuclei were stained with DAPI (blue). Scale bar, 10 μm. **f** Fluorescence recovery after photobleaching (FRAP) assay of the droplets formed by EGFP-MEX3A-FL and EGFP-MEX3A-IDRs in DLD-1 cells. Top, representative images. Scale bar, 500 nm. Bottom, quantification of fluorescence intensity recovery after photobleaching. **g** Kyoto Encyclopedia of Genes and Genomes (KEGG) pathway enrichment analysis showing the proteins interacting with MEX3A involved in the RNA catabolic process, RNA nonsense-mediated decay, and translation. The size of the dot indicates the number of genes per pathway. **h** Western blotting of MEX3A-IP assay showing the binding between MEX3A and the PBs proteins in 293 T (left) and DLD-1 cells (right). **i** IF assay showing that MEX3A was colocalized with the PBs proteins MOV10, PABP1, and UPF1 in DLD-1 cells. Nuclei were stained with DAPI (blue). Scale bar, 10 μm

The MEX3A protein appears to contain several intrinsically disordered regions (IDRs) predicted by online IUPred2A tool (http://iupred2a.elte.hu) (Fig. 2a) which are known to result in the formation of phase separation droplets.^25,26^ To investigate whether MEX3A undergoes LLPS through IDRs, we constructed MEX3A full-length and two IDRs truncated mutant plasmids (Fig. 2b). We first purified full-length recombinant MEX3A. MEX3A solutions were transparent at 4 °C and turned opaque gradually as the temperature increased to 37 °C (Fig. 2c). Under phase-contrast microscopy, micrometer-sized droplets spontaneously increased in size and number with elevated concentrations of MEX3A and NaCl in solutions (Fig. 2d). Truncating the IDRs showed remarkably increased droplets size and number, while deleting the IDRs made it difficult to observe droplets compared to the full-length MEX3A (Fig. 2c, d). These findings align with the reported view that phase separation exhibits temperature, protein, and salt concentration dependence.^27^

To assess whether MEX3A and truncations also form liquid-like droplets in live cells, we ectopically expressed EGFP–MEX3A in CRC cells and observed that EGFP–MEX3A exhibited discrete puncta distribution in the cytoplasm (Fig. 2e, Supplementary Fig. S3a). Using the fluorescence recovery after photobleaching (FRAP) assay, we observed that the fluorescent signal of EGFP-MEX3A punctum recovered 70% within 6 min after photobleaching (Fig. 2f, Supplementary Fig. S3b, Supplementary Movie S1-2), indicating that MEX3A can exchange between the droplet aggregation and the surrounding cytosol. Moreover, cells transfected with EGFP-MEX3A-IDRs showed massive droplet-like condensates in the cytoplasm, which gradually fused over time and recovered 90% within 6 min after photobleaching. In contrast, cells transfected with EGFP-MEX3A-IDRs△ presented limited cytoplasmic puncta that were unable to fuse and recover after photobleaching (Fig. 2e, f, Supplementary Fig. S3a, b, Supplementary Movie S3–6). Furthermore, upon the IDRs deletion, the increased subcutaneous tumor growth by MEX3A overexpression was attenuated (Supplementary Fig. S3c, d). Collectively, these results suggest that the IDRs of MEX3A are required for its LLPS and function in CRC tumorigenesis.

### MEX3A physically interacts with PBs components

To explore the underlying mechanisms of MEX3A in promoting CRC pathogenesis, we performed co-immunoprecipitation (co-IP) assay and mass spectrometry analysis to screen MEX3A-interacting partners in 293T cells (Supplementary Fig. S4a). Intriguingly, we filtered several proteins mainly involved in the RNA catabolic process, RNA nonsense-mediated decay and translation, among which have been previously identified as PBs-associated RBPs, including MOV10, UPF1, DDX6, and PABP1 et al. (Fig. 2g, Supplementary Table S3). Co-IP assay further validated the association of MEX3A with MOV10, PABP1, and UPF1 in 293T and CRC cells (Fig. 2h, Supplementary Fig. S4b). By performing immunofluorescence (IF) assay, we confirmed that exogenously expressed EGFP-MEX3A was concentrated in cytoplasmic MOV10- or PABP1- or UPF1-containing foci (Fig. 2i, Supplementary Fig. S4c). Consistent with the previous discovery for MCF7 cells that MEX3A localizes to DCP1a-containing PBs,^28^ our findings strongly support that MEX3A protein is one component of PBs.

### MEX3A forms a complex with circMPP6 in PBs

**Fig. 3:**
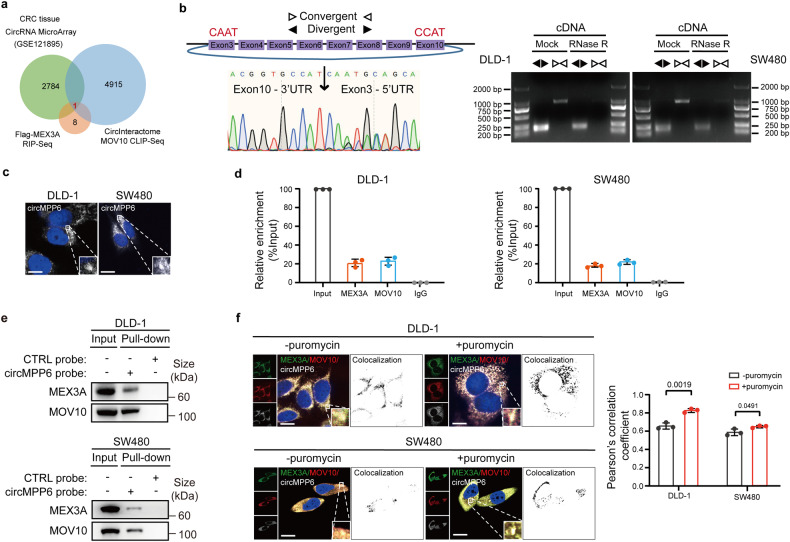
MEX3A forms a complex with circMPP6 in PBs. **a** Venn diagram showing the overlap between CRC tissue CircRNA MicroArray, RIP-Seq analysis of Flag-MEX3A, and CLIP-Seq analysis of MOV10 by CircInteractome (https://circinteractome.nia.nih.gov/). **b** The genomic locus of circMPP6. Left, Sanger sequencing showing the back splicing junction site of circMPP6. Right, PCR products with or without RNase R pre-treatment showing circularization of circMPP6 in DLD-1 and SW480 cells. cDNA, complementary DNA. **c** RNA fluorescence in situ hybridization (RNA FISH) assay showing the distribution of circMPP6 in DLD-1 and SW480 cells. Nuclei were stained with DAPI (blue). Scale bar, 10 µm. **d** qRT–PCR analysis of RIP assay verifying the binding of circMPP6 to MEX3A and MOV10 in DLD-1 and SW480 cells. IgG antibody served as the negative control. **e** Western blotting of circMPP6 Pull-down assay showing the specific association of MEX3A and MOV10 protein with circMPP6 in DLD-1 and SW480 cells. **f** IF-FISH assays showing that the colocalization of MEX3A/MOV10/circMPP6 was increased after incubation with 20 μg/mL puromycin for 4 h in DLD-1 and SW480 cells. Left, representative images. Nuclei were stained with DAPI (blue). Scale bar, 10 µm. Right, Pearson’s correlation coefficient analysis. Data are represented as mean ± S.D. from three independent experiments (**d**, **f**). The *P* value was determined by a two-tailed unpaired Student’s *t* test (**f**)

Given that PBs arise as a consequence of high local concentrations of key proteins and RNAs, we wondered whether small RNAs participate in the MEX3A-guided PBs homeostasis. To further identify the potential MEX3A-binding circRNAs, we performed RIP assay and small RNA-Seq analysis. From previous CircRNA MicroArray data of CRC tissues (GSE121895),^18^ 2784 circRNAs were upregulated (fold change >2). Among these, only two circRNAs were predicted to bind to MEX3A (Input >1, fold change >2). Subsequently, we screened MOV10-binding circRNAs from published RBP crosslinking-immunoprecipitation and high-throughput sequencing (CLIP-Seq) datasets in CircInteractome (https://circinteractome.nia.nih.gov/) and identified circ_0001686 for further investigation (Fig. 3a).

Referring to the human reference genome (GRCh37/hg19), circ_0001686 is mapped to the exons 3–10 regions within the *MPP6* locus, and thus termed as circMPP6. Then we conducted a series of experiments to characterize circMPP6. Sanger sequencing after RT-PCR with divergent primers verified the specific back-splicing junction site (Fig. 3b). Following RNase R and Actinomycin D treatment assays demonstrated that circMPP6 was more resistant to RNase R and more stable than the parental *MPP6* mRNA (Supplementary Fig. S5a, b). Furthermore, nuclear and cytoplasmic fractionation assay revealed that circMPP6 was predominantly localized in the cytoplasm (Supplementary Fig. S5c). Notably, fluorescence in situ hybridization (FISH) examination presented the cytoplasmic punctate distribution of circMPP6 (Fig. 3c).

We examined the expression of circMPP6 in 30 CRC tissues compared to paired normal tissues and found that the circRNA was significantly increased in CRC tissues (Supplementary Fig. S5d). Using RIP and RNA Pull-down assays, we confirmed that circMPP6 interacted with MEX3A and MOV10 (Fig. 3d, e, Supplementary Fig. S5e), a core component of PBs. Further, we incubated CRC cells in the absence or presence of puromycin, a stimulant of PBs formation. From immunofluorescence and RNA fluorescence in situ hybridization (IF-RNA FISH) assays, we detected that MEX3A, MOV10, and circMPP6 presented cytoplasmic colocalization under physiological conditions. Strikingly, the treatment of puromycin accelerated the recruitment of circMPP6 and MEX3A protein into cytoplasmic foci which stained positive for the MOV10 marker (Fig. 3f). Moreover, we also tested whether circMPP6 has similar function to MEX3A in CRC cells. As indicated by colony formation and Transwell migration assays, circMPP6 overexpression greatly increased cell proliferation and migration abilities of CRC cells (Supplementary Fig. S6a–c). As expected, in subcutaneous xenograft model, circMPP6 overexpression substantially enhanced the tumorigenicity and tumor growth of CRC cells (Supplementary Fig. S6d), which indicates the potential mediator role of circMPP6 for MEX3A. These data reveal the formation of the MEX3A/circMPP6 complex, and indicate a close relationship between the MEX3A/circMPP6 complex and the PBs formation in CRC cells.

### circMPP6 facilitates PBs aggregation by strengthening MEX3A-PBs components’ interaction

We next explored how circMPP6 regulates the function of MEX3A. Western blotting showed that circMPP6 knockdown did not affect MEX3A expression (Supplementary Fig. S7a). MEX3A is known to involve in protein ubiquitylation.^29^ Since circRNAs can modulate proteins degradation by acting as a scaffold,^30,31^ we wondered if the MEX3A/circMPP6 complex affects the protein expression of certain PBs components. Western blotting showed that the expression of PBs components remained unaffected upon MEX3A and/or circMPP6 knockdown (Supplementary Fig. S7a), excluding the possibility that the MEX3A/circMPP6 complex functions in this way.

**Fig. 4:**
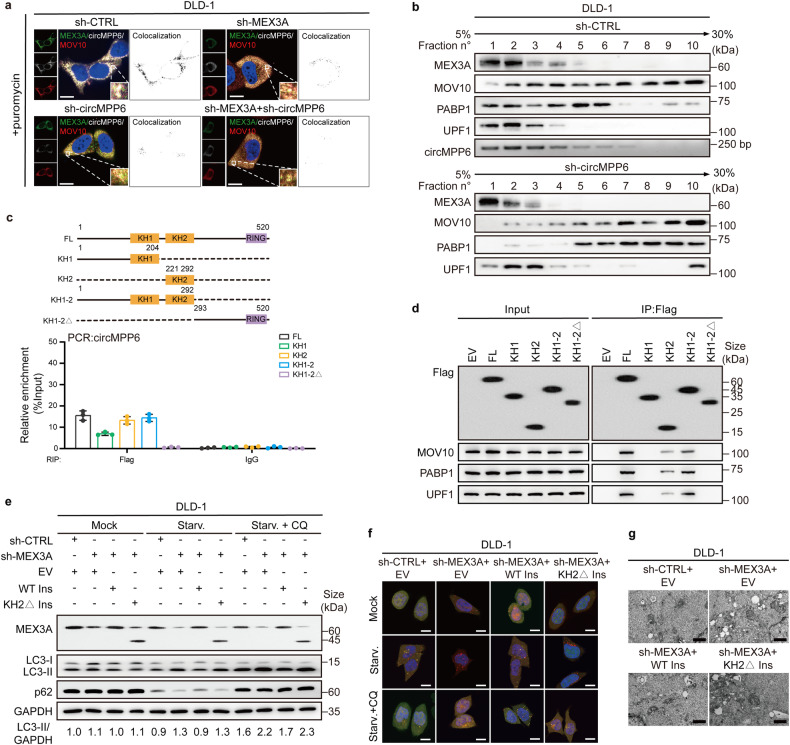
circMPP6 facilitates PBs aggregation by strengthening MEX3A-PBs components' interaction. **a** IF-FISH assays showing that the colocalization of MEX3A/circMPP6/MOV10 was decreased upon knockdown of MEX3A and/or circMPP6 after treatment with puromycin in DLD-1 cells. Nuclei were stained with DAPI (blue). Scale bar, 10 µm. **b** Sucrose gradient fraction analysis of a whole-cell lysate from control (top) and circMPP6-knockdown (bottom) DLD-1 cells. **c** Top, the schematic diagram representing the Flag-MEX3A truncations. Bottom, qRT–PCR analysis of RIP assay showing the interactions between circMPP6 and each truncation. **d** Western blotting of Flag-IP assay in DLD-1 cells showing the interactions between PBs proteins and each truncation. **e** Western blotting showing the changes of LC3 conversion and p62 expression upon MEX3A knockdown could be rescued by overexpression of WT, but not the mutant MEX3A in DLD-1 cells. Representative images of the autophagic flux with the mRFP-GFP-LC3 reporter (**f**), and ultrastructural autophagosomes and/or autolysosomes (**g**) illustrating that MEX3A knockdown-induced autophagy could be counteracted by overexpression of WT, but not the mutant MEX3A in DLD-1 cells. Scale bar, 10 μm (**f**), 500 nm (**g**). Data are represented as mean ± S.D. from three independent experiments (**c**)

We then focused on the accumulations of PBs. IF-RNA FISH assays showed that in puromycin pre-condition, the absence of MEX3A and/or circMPP6 induced P bodies dissociation, whilst both MEX3A and circMPP6 fluorescent-tagged fusions were presented in the cytoplasm (Fig. 4a, Supplementary Fig. S7b). Thus, we hypothesized that the MEX3A/circMPP6 complex may modulate PBs assembly. To determine whether circMPP6 affects the binding of MEX3A with the three PBs-related proteins, co-IP was performed using control or circMPP6-knockdown CRC cells. Notably, when equal amounts of MEX3A proteins were pulled down, silencing of circMPP6 decreased the abundance of PBs-related proteins, including MOV10, PABP1, and UPF1, in MEX3A immunoprecipitated fractions. Moreover, treatment with RNase A (an RNA endonuclease degrading circRNAs) severely weakened these interactions (Supplementary Fig. S7c). We then assumed that circMPP6 may reinforce the interaction of MEX3A, MOV10, PABP1, and UPF1 to form multiple complexes. To validate this hypothesis, we separated the lysates of either control or circMPP6-knockdown CRC cells by sucrose gradient sedimentation. Western blotting was used to examine the sedimentation profile of each protein for individual sucrose gradient fractions. MEX3A, MOV10, PABP1, and UPF1 showed a comparable distribution ranging from fraction 1 to fraction 4, indicating the formation of a multi-protein complex in CRC cells. Moreover, the distribution of circMPP6 visualized by agarose gel electrophoresis was also enriched in fraction 1 to 4. In the extract derived from circMPP6-knockdown cells, we detected a very low level of MEX3A-PBs components complex in fraction 3 and 4; MEX3A displayed a high level in fraction 1; MOV10, PABP1, and UPF1 migrated to the higher molecular weight fractions (Fig. 4b, Supplementary Fig. S7d). Collectively, these results suggest the accumulation of MEX3A with the protein components of PBs dependent on circMPP6.

We further sought to determine whether circMPP6 serves as a scaffold to bring MEX3A and the three proteins together, promoting PBs aggregation. To identify the domain of MEX3A bound to circMPP6, we constructed MEX3A mutants by individually truncating heterogeneous nuclear ribonucleoprotein K homology (KH) domains, a conserved region of 65–70 amino acids that interacts with RNA.^32^ RIP assay showed that both the KH1 and KH2 domains of MEX3A are responsible for the MEX3A/circMPP6 complex formation (Fig. 4c, Supplementary Fig. S8a). Further co-IP investigation revealed that MEX3A recruited these three PBs-related factors via its KH2 domain (Fig. 4d, Supplementary Fig. S8b). Overall, we confirmed that the KH2 domain of MEX3A bound with circMPP6 is required for recruiting PBs components.

We subsequently investigated if the KH2 domain contributes to MEX3A-mediated CRC aggressiveness. Rescue assays showed that exogenous expression of MEX3A wild-type (WT), but not MEX3A KH2 deletion, distinctly reversed the pro-autophagic effect of silencing MEX3A (Fig. 4e–g, Supplementary Fig. S9a-c). Consistently, colony formation and Transwell migration assays demonstrated that enforced expression of MEX3A WT, but not MEX3A KH2 deletion reversed the proliferation and metastasis-inhibitory effects of silencing MEX3A (Supplementary Fig. S9d, e). These data emphasize the crucial role of the KH2 domain in MEX3A-mediated CRC aggressiveness.

To better characterize the binding of circMPP6 with MEX3A, we re-analyzed the RIP-Seq data and identified the CACU RNA sequence as a MEX3A-binding motif **(**Supplementary Fig. S10a). Surprisingly, there is a CACU motif in the exon 10−exon 3 junction sequence of circMPP6. We next synthesized a biotin-labeled circMPP6 WT probe and mutant probe of which the CACU motif changed to GUGC (Supplementary Fig. S10b). RNA Pull-down assay showed that MEX3A was immunoprecipitated by the circMPP6 WT probe but not the control or mutant probe (Supplementary Fig. S10c). Consistently, in vitro RNA electrophoretic mobility shift assay (RNA-EMSA) verified that the CACU motif within circMPP6 is responsible for its binding with MEX3A (Supplementary Fig. S10d). Further phenotype assays validated the essential role of CACU motif in circMPP6 interaction with MEX3A and its function in CRC pathogenesis (Supplementary Fig. S10e-i). Therefore, our results imply that circMPP6 facilitates maintaining the PBs aggregation by affecting the collaboration of MEX3A-PBs components. Together these data provide, for the first time, a link between RBP/circRNA complex and PBs.

### The MEX3A/circMPP6 complex promotes *PDE5A* mRNA decay

**Fig. 5:**
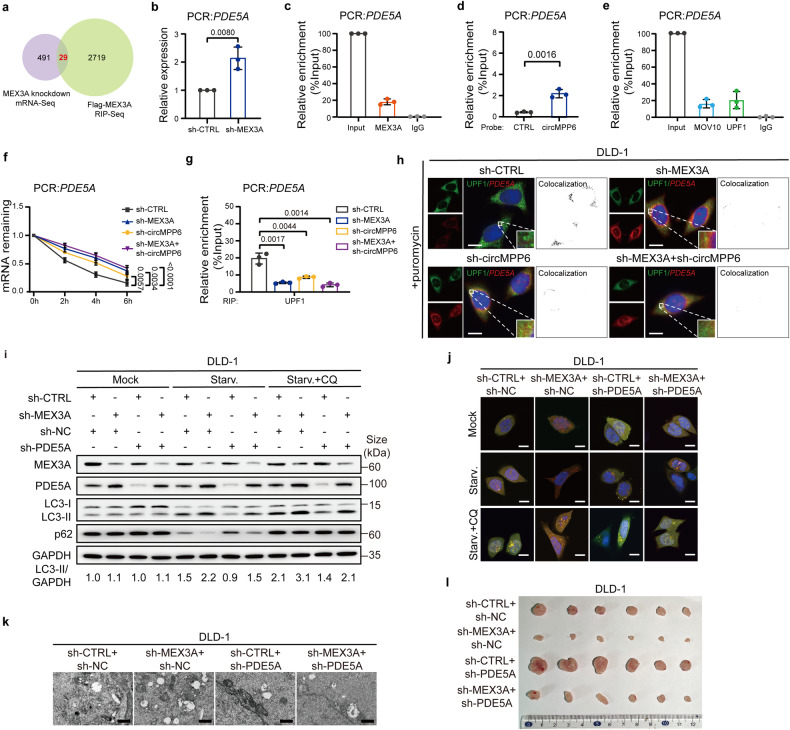
The MEX3A/circMPP6 complex promotes *PDE5A* mRNA decay and modulates CRC autophagy through the PDE5A pathway. **a** Venn diagram showing the overlap of MEX3A-interacting mRNAs identified by Flag-MEX3A RIP-Seq and MEX3A knockdown mRNA-Seq data. **b** qRT–PCR analysis for the expression of *PDE5A* in MEX3A-knockdown DLD-1 cells. **c** qRT–PCR analysis of RIP assay showing the interaction of *PDE5A* with MEX3A in DLD-1 cells. IgG served as the negative control. **d** qRT–PCR analysis of RNA Pull-down verifying the interaction of *PDE5A* with circMPP6 in DLD-1 cells. **e** qRT–PCR analysis of RIP analysis verifying the interaction of *PDE5A* with MOV10 or UPF1 in DLD-1 cells. **f** qRT-PCR analysis for the expression of *PDE5A* after treatment with Actinomycin D at indicated time points in indicated DLD-1 cells. **g** qRT–PCR analysis of RIP assay showing the association of *PDE5A* with UPF1 in indicated DLD-1 cells. **h** IF-FISH assays showing the colocalization of UPF1/*PDE5A* and the fluorescence intensity of *PDE5A* in indicated DLD-1 cells after treatment with puromycin. Nuclei were stained with DAPI (blue). Scale bar, 10 μm. **i** Western blotting showing the changes of LC3 conversion and p62 expression upon MEX3A knockdown could be rescued by PDE5A silencing. Representative images of the autophagic flux with the mRFP-GFP-LC3 reporter (**j**), and ultrastructural autophagosomes and/or autolysosomes (**k**) illustrating that MEX3A knockdown-induced autophagy could be counteracted by PDE5A silencing. Scale bar, 10 μm (**i**), 500 nm (**j**). **l** The subcutaneous xenograft model showing decreased tumor formation upon MEX3A knockdown was rescued by silencing PDE5A in BALB/c nude mice (*n* = 6). Data are represented as mean ± S.D. from three independent experiments (**b**–**g**). The *P* value was determined by a two-tailed unpaired Student’s *t* test (**b**, **d**, **g**) or a two-way ANOVA (**f**)

To profile the downstream targets of MEX3A, mRNA-Seq in control or MEX3A-knockdown DLD-1 cells were applied. Among 491 differentially expressed mRNAs upon MEX3A knockdown (fold change > 2), we cross-referenced MEX3A RIP-Seq data and identified 29 mRNAs bound to MEX3A (Fig. 5a). Next, we selected ten recurrently and significantly dysregulated mRNAs (occurrent in both TCGA patient samples and CRC cell lines) as validation candidates. Through further qRT-PCR examinations followed by Flag-MEX3A-RIP assay (Fig. 5b, c, Supplementary Fig. S11a, b), we identified that *PDE5A*, *SENP7*, and *TTLL7* mRNAs were regulated and bound by MEX3A. The interaction between circMPP6 and these three mRNAs was verified by RNA Pull-down assay (Fig. 5d, Supplementary Fig. S11c). Moreover, the PBs structure components, MOV10 and UPF1, presented the ability to recruit the mRNAs (Fig. 5e, Supplementary Fig. S12a), consistent with the analysis from published CLIP-Seq datasets in ENCORI (https://rnasysu.com/encori/). Moreover, significantly enhanced *PDE5A*, *SENP7*, and *TTLL7* mRNA stability was observed in CRC cells following MEX3A/circMPP6 knockdown (Fig. 5f, Supplementary Fig. S12b). These data indicate that the MEX3A/circMPP6 complex mediated PBs aggregation affects mRNAs degradation. Considering MEX3A inhibits the autophagy progression, *PDE5A* was identified as the potential target of MEX3A according to the reported literature.^33,34^ Consistent with mRNA stability, PDE5A protein was also increased upon MEX3A/circMPP6 depletion (Supplementary Fig. S12c).

As UPF1, a part of PBs-related machinery, triggers mRNA decay,^35^ we speculated that the MEX3A/circMPP6 complex may recruit UPF1 to initiate *PDE5A* mRNA degradation. To validate this hypothesis, we performed RIP assay and found that the MEX3A/circMPP6 knockdown significantly decreased the *PDE5A* mRNA-binding ability to UPF1 (Fig. 5g, Supplementary Fig. S13a). Further IF-RNA FISH assays showed that *PDE5A* mRNA colocalized with UPF1 in the cytoplasm under physiological conditions. Notably, the puromycin treatment diminished the fluorescence intensity of *PDE5A* mRNA (Supplementary Fig. S13b). In the absence of MEX3A/circMPP6, the colocalization of the UPF1/*PDE5A* complex decreased and the fluorescence intensity of *PDE5A* mRNA increased (Fig. 5h, Supplementary Fig. S13c). These findings demonstrate that the accumulation of a MEX3A/circMPP6 complex-dependent interaction between *PDE5A* and UPF1 enhances *PDE5A* mRNA degradation.

### MEX3A modulates CRC autophagy through the PDE5A pathway

To determine whether MEX3A regulates CRC malignancy in a PDE5A signaling pathway, we performed rescue assays by transfecting PDE5A shRNA into control and MEX3A-knockdown CRC cells. Inhibiting PDE5A distinctly suppressed the autophagy program of CRC and even reversed the pro-autophagic effect of silencing MEX3A **(**Fig. 5i-k, Supplementary Fig. S14a–c). Consistently, colony formation and Transwell migration assays demonstrated that PDE5A suppression prominently reversed the proliferation and metastasis-inhibitory effects of MEX3A-silenced CRC cells (Supplementary Fig. S15a, b). The xenotransplant model demonstrated that the decreased tumorigenicity of MEX3A-knockdown cells was largely rescued by PDE5A inhibition in vivo (Fig. 5l, Supplementary Fig. S15c).

We next detected the in vivo expression levels of PDE5A in subcutaneous tumors of nude mice injected with control or MEX3A-knockdown or MEX3A-overexpression CRC cells. We observed a reversed immunostaining pattern of PDE5A in transplanted tumors compared to that of MEX3A (Supplementary Fig. S15d, e). These findings indicate that the oncogenic effects of MEX3A in CRC depend on the PDE5A pathway.

### MEX3A/circMPP6-PDE5A axis is correlated with CRC outcomes

**Fig. 6:**
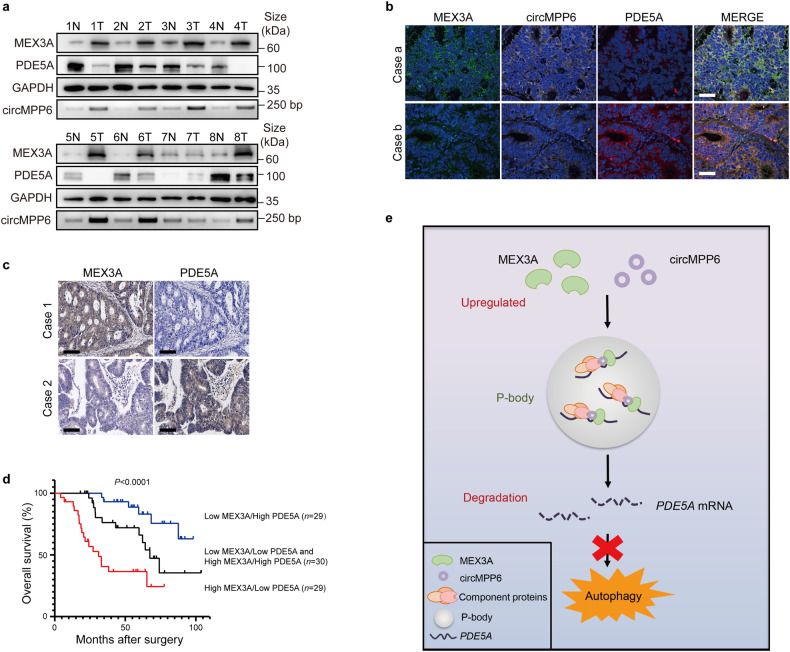
MEX3A/circMPP6-PDE5A axis is clinically correlated with CRC outcomes. **a** Western blotting of MEX3A and PDE5A protein expression and qRT-PCR analysis of circMPP6 RNA expression in 8 pairs of matched CRC tumor (T) and adjacent noncancerous tissues (N). GAPDH was used as the loading control. **b** Representative RNA-FISH and immunofluorescence staining images of circMPP6 and PDE5A in two CRC tissues with high (the first row) or low MEX3A expression (the second row). Nuclei were stained with DAPI (blue). Scale bar, 100 μm. **c** Representative IHC images of MEX3A and PDE5A protein staining in CRC tissue microarrays from SYSUCC. Scale bar, 100 μm. **d** Kaplan-Meier analysis of OS for CRC patients from SYSUCC cohorts (*n* = 88) indicating that high MEX3A and low PDE5A were associated with a poor prognosis. The *P* value was determined by a Log-rank test. **e** A proposed model demonstrating that the MEX3A/circMPP6 complex inhibits CRC cell autophagy by mediating PBs-dependent *PDE5A* mRNA decay

To further investigate the clinical significance of MEX3A modulation in CRC patients, we assessed the RNA expression levels of *MEX3A*, circMPP6, and downstream *PDE5A* in CRC tissues from TCGA and SYSUCC cohorts. Similar results were obtained that the expression of *PDE5A* was negatively correlated with the levels of *MEX3A* transcripts and circMPP6 (Supplementary Fig. S16a). Next, we compared another 8 matched CRCs and adjacent noncancerous tissues and found the protein expression of MEX3A and RNA expression of circMPP6 were frequently upregulated whereas PDE5A was downregulated (Fig. 6a). Moreover, RNA-FISH and double immunofluorescence staining showed a negative relevance between the expression of PDE5A and that of MEX3A/circMPP6 in CRC tissues (Fig. 6b, Supplementary Fig. S16b). IHC staining analysis revealed the reverse immunostaining intensities between PDE5A and MEX3A expression in most CRC tissue specimens (Fig. 6c, Supplementary Fig. S16c). Notably, the Kaplan-Meier survival analysis of patients classified into subgroups with either low or high expression of MEX3A/PDE5A proteins illustrated that CRC patients exhibiting high MEX3A and low PDE5A had the worst OS in our cohorts (Fig. 6d). Collectively, these results support that MEX3A upregulation significantly attenuates the expression of PDE5A, thus exerting a critical role in CRC aggressiveness and patients prognosis.

## Discussion

The present study demonstrated that MEX3A is frequently augmented in CRC patients and positively correlated with poor prognosis. MEX3A promotes CRC proliferation and migration, and suppresses autophagy program. In addition, MEX3A undergoes LLPS in the cytoplasm, adding a layer of complexity to its cellular dynamics. Mechanistically, MEX3A interacts with circMPP6 to produce an RBP-circRNA complex that facilitates the recruitments of PBs and thus enhances *PDE5A* mRNA decay, which promotes aggressiveness in CRC cells, as depicted in Fig. 6e. Clinically, the coexistence of high MEX3A and low PDE5A in CRC patients predicts the poorest survival outcome. These findings elucidate a previously unrecognized biological significance and molecular mechanism of the MEX3A/circMPP6 complex underlying the CRC pathogenesis, providing novel prognostic markers and therapeutic targets for the CRC personalized treatment.

The disruption of LLPS has recently been documented to result in oncogenesis.^36^ LLPS condensates diverse molecules, such as proteins and nucleic acid, into membrane-less organelles documented as SGs and PBs.^37^ These phase-separated aggregations play an important part in epigenetic regulation by gathering signaling activators. PBs are considered as the mRNA storage or degradation center, but their contributions to tumorigenesis remain to be deciphered. A current study has shown that the dysregulation of SYK localized in PBs affects PBs formation during the EMT development in breast cancer.^38^ Abro1 and FANCD2 have been reported to disrupt the formation of replication stress-elicited PBs, which contribute to promoting the innate immune response following persistent replication stress in the osteosarcoma cell line U2OS.^39^ Our study provides the first evidence that MEX3A accumulates into cytoplasmic droplets formed through LLPS and recruits several structure containing factors to assemble PBs in CRC cells. On the other hand, certain RNA chaperones may be associated with PBs.^40^ Both m6A and miRNA, the known regulators of post-transcription modification, can urge mRNA transcripts to be stored or degraded in the PBs.^41,42^ Cancer cells may alter the decay machinery and profit from those mRNA decay shredders assembled in PBs to deviate gene regulation by elevating the expression level of oncogenes and attenuating the expression of the tumor suppressor genes. The functional mechanisms of circRNAs largely depend on the subcellular locations. Cytoplasmic circRNAs usually function by sponging miRNAs^15^ or interacting with proteins.^18,19^ In the current study, circMPP6 was predominantly located in the cytoplasm and identified as an interactor of MEX3A. Additionally, it was worth noting that circMPP6 and MEX3A presented the cytoplasmic punctate colocalization in CRC cells. circMPP6 acted as a scaffold to facilitate the interaction of MEX3A with three PBs-related proteins including UPF1, a well-established RNA decay factor concentrated in PBs,^35^ leading to PBs aggregations and thus mediating the mRNA fate. To our knowledge, this is the first reported circRNA engaging in PBs homeostasis.

So far, only a few studies have reported that circRNAs function by interaction with RBPs in cancer, thus stabilizing mRNAs and increasing translation. circNSUN2 enhances the stability of *HMGA2* mRNA to promote CRC metastasis progression by forming a circNSUN2/IGF2BP2/*HMGA2* RNA-protein ternary complex.^18^ circMYBL2 exacerbates acute myeloid leukemia by strengthening the interaction between PTBP1 and *FLT3* mRNA, thus promoting translation.^19^ Here, we unveiled a potent mechanism through which circMPP6 can facilitate *PDE5A* mRNA decay by strengthening the interaction between MEX3A and UPF1, a crucial component of PBs aggresome processing. Hence, in the realm of circRNA regulation, we uncovered a unique function whereby circRNAs can facilitate mRNA degradation by recruiting RBPs into cytoplasmic PBs, expanding our understanding of circRNAs functions in RNA metabolism. Notably, from the aspect of MEX3A, we unraveled a regulatory mechanism that governs mRNA decay in a circRNA-mediated PBs-dependent manner. To date, this is the first investigation into the MEX3A-circRNAs network in cancer.

Autophagy is an alternative process for maintaining cellular homeostasis and plays a complex and context-dependent role in carcinogenesis.^43^ In general, autophagy has evolved to cope with intracellular and extracellular stress, thereby determining the survival and death of cells under different circumstances.^44^ Some researchers have linked autophagy to the repression of CRC tumorigenesis; however, other studies have shown that autophagy is positively correlated with CRC advancement. A previous study demonstrated that Ube2v1 promotes the growth and metastasis of CRC by epigenetically suppressing autophagy.^45^ An additional report showed that PKCλ/ι activation suppresses both autophagy and IFN signaling, and thus prevents immunoevasion in CRC.^46^ These findings imply that autophagy may have differential influences at distinct stages of tumorigenesis. Recent research has revealed the formation of a miRNA/MEX3A complex at the autophagic vesicle surface. Deletion of MEX3A or impairment of endothelial autophagy diminishes nuclear import of miR-126-5p, accelerates endothelial apoptosis, thereby contributing to atherosclerosis escalation.^7,47^ Here, we identified a vital role of MEX3A in tumor growth and metastasis of CRC in vivo and in vitro. Noteworthily, our study links the MEX3A-binding mRNAs with autophagy pathways in CRC, suggesting these molecular cooperative interactions contribute to the autophagy program in CRC. We further found that MEX3A modulates the expression of autophagy receptors and blocks autophagic flux in CRC cells, supporting the emerging concept of MEX3A as a selective regulator of autophagy in CRC. As reported in the literature, PDE5A tends to play a particularly interesting role in cell autophagy. Icariin diminishes the enzymatic activity of PDE5A and significantly prevents mitochondrial dysfunction and the autophagy process, thereby leading to cardioprotective effects.^34^ In addition, the principal active constituents of SMYAD retrain autophagy by inhibiting PDE5A, thereby modulating the AKT/mTOR/ULK1 pathway in isoproterenol-induced heart failure.^33^ Here, we identified PDE5A as one of the potent targets of the MEX3A/circMPP6 complex during CRC progression and proposed a crucial PBs-dependent mechanism for *PDE5A* mRNA degradation in CRC. Our data elucidated that the function of MEX3A in suppressing CRC cell autophagy relies on the PDE5A pathway, providing the first evidence of PDE5A as a critical regulator in cancer cell autophagy.

In summary, data from the current study characterized, for the first time, a novel function of the MEX3A/circMPP6 complex in the modulation of PBs-mediated mRNA decay associated with CRC pathogenesis. Therefore, our findings have uncovered a brand-new mechanism underlying MEX3A-induced tumor progression and might hint an auxiliary target for advancing CRC pharmacological therapies.

## Materials and methods

### Human specimens

This study has been approved by the Institutional Review Board of Sun Yat-sen University Cancer Center (SYSUCC, Guangzhou, China) and Guangdong Provincial People’s Hospital (GDPH, Guangzhou, China). Informed consent was acquired from the participating patients before the research began.

For copy number examinations and Western blotting, ten primary CRC tissues and adjacent normal tissues were collected between 2010 and 2015 at SYSUCC. For IHC analysis, the tissue microarray containing samples from 88 CRC patients was obtained from SYSUCC between 2010 and 2015. For IF-RNA FISH staining, 12 tumor samples were derived from CRC patients at SYSUCC in 2023. The selection criteria for CRC cases included clear imaging and pathological diagnosis, complete follow-up data, and no previous local or systemic treatment. Tumor grade and stage were determined according to the WHO criteria and the eighth edition of the TNM classification of the UICC (2016).

### Animal experiments

All the animal experiments were approved by SYSUCC Animal Care and Use Committee.

The female BALB/c nude mice from Vital River Laboratory (Beijing, China) were housed under specific pathogen-free conditions. The CRC cells were transfected with lentiviral vectors for stable knockdown or overexpression of MEX3A, PDE5A, or circMPP6. Treated CRC cells (2 × 10^6^ CRC cells in 0.1 mL of serum-free culture medium) were subcutaneously injected into the flank of 4-week-old nude mice (*n* = 6/group). After 4 weeks, the nude mice were sacrificed, and the subcutaneous tumors were excised and weighed.

### mRFP-GFP-LC3 reporter assay

Cells were transfected with the mRFP-GFP-LC3 lentivirus. With the construct, yellow fluorescence (mRFP+/GFP+) indicates autophagosomes, and red fluorescence (mRFP+/GFP-) represents autolysosomes, which was assessed by confocal fluorescence microscopy (OLYMPUS FV1000, Japan).

### Protein purification

The GST-tagged MEX3A full-length or truncated plasmids in the pGEX-6P-1 vector were transformed into BL21 (DE3) competent *E.coli* (TIANGEN, Beijing, China, #CB105). The *E.coli* cultured at OD600 of 0.6 were induced with 0.2 mM of IPTG (Solarbio, Beijing, China, #I8070-1) for 12 h at 16 °C to express recombination proteins. GST-tagged proteins were then purified using the GST-tag Protein Purification Kit (Beyotime, Shanghai, China, #P2262). Purified proteins were validated by Coomassie blue staining.

### Droplet formation assay

Droplet formation assay was performed in 20 mM HEPES pH 7.4, 300 mM KCl, 6 mM MgCl_2_, 1 mM DTT, and 10% PEG8000 with the indicated protein and NaCl concentrations. After incubation at 37 °C for 90 min, droplets were observed by phase-contrast microscopy (Nikon ECLIPS Ti-2, Japan).

### Fluorescence recovery after photobleaching assay (FRAP)

Cells were transfected with different EGFP-tagged MEX3A truncated plasmids. FRAP assay was conducted on a Zeiss LSM880 laser-scanning confocal microscope (Germany) with a fast airy scan. The fluorescence was bleached with 90% of laser power for 30 s with 488 nm laser, and the recovery of fluorescence was tracked for 6 min at a 0.5-s interval. The data were normalized against the overall photobleaching for the EGFP fluorescence.

### Immunoprecipitation (IP) assay

IP assay was conducted as previously described.^29^ The immunoprecipitated proteins were evaluated by Western blotting.

### Mass spectrometry (MS) analysis

Silver staining was performed by using the Fast Silver Stain Kit (Beyotime, Shanghai, China, #P0017S). While protein identification, quantification and analysis were done by FitGene (Guangzhou, China) based on MS. Proteins with area ratio (MEX3A/CTRL) > 2, unique peptides > 2 were shown in Supplementary Table S3.

### RNA-binding protein immunoprecipitation (RIP)

RIP assay was conducted by using Magna RIP RNA-Binding Protein Immunoprecipitation Kit (Millipore, MA, USA, #17-704). The beads-bound RNA was analyzed by RNA-Seq or qRT-PCR analysis. RNA-Seq was conducted by Geneseed Technology (Guangzhou, China).

### RNA Pull-down

The biotin-labeled circMPP6 probe was incubated with streptavidin-coated magnetic beads (Invitrogen, California, USA, #65001), according to the manufacturer’s protocols. The probe sequence is shown below:

circMPP6 junction probe (Umine-Bio, Guangzhou, China):

5′-biotin-GCUGCAUUGAUGGCACCGUAGUUCCAAAUCUAGUGGGA-Cy3-3′

### RNA fluorescence in situ hybridization (FISH)

The assay was performed with Fluorescent in Situ Hybridization Kit (GenePharma, Suzhou, China, #F0539). The images were acquired and analyzed on the OLYMPUS FV1000 confocal microscopy system. The probe sequences are shown below:

circMPP6 probe (Umine-Bio, Guangzhou, China):

5′-biotin-GCUGCAUUGAUGGCACCGUAGUUCCAAAUCUAGUGGGA-Cy3-3′

*PDE5A* probe (Umine-Bio, Guangzhou, China):

5′-biotin-UCUUCUCCUGCUGUUCUGCAAGGGCCUGCCAUUUCUGCCUGU

UCUUUC-Cy5-3′

### Sucrose gradient sedimentation

Cells were lysed with lysis buffer (20 mM HEPES pH 7.4, 150 mM NaCl, 10 mM KCl, 2 mM MgCl_2_, 2 mM DTT, 0.5 mM EDTA, 0.5% NP-40, 0.1% PMSF and 1× Protease Inhibitor Cocktail). The cell extracts were loaded on top of a linear 5–30% (w/v) sucrose gradient and centrifuged for 12 h at 4 °C, 36,000 rpm using a Beckman SW41 rotor (OptimaTM L-100 XP, USA). Then the sucrose gradient was fractionated into ten fractions and analyzed by Western blotting or qRT-PCR.

### Electrophoretic mobility shift assay (EMSA)

Biotin-labeled RNA probes were synthetized from Tsingke Biotech (Beijing, China). The assay was performed with LightShift™ Chemiluminescent RNA EMSA Kit (Thermo Fisher Scientific, Waltham, USA, #20158).

### mRNA-Seq

The mRNA-Seq was conducted by LC-Bio Technology (Hangzhou, China) according to the method described previously.^48^ The differentially expressed mRNAs were selected with fold change >2 and *P* value < 0.05 by R package edgeR.

### Immunohistochemistry (IHC) staining

The slides were deparaffinized, subjected to antigen retrieval with EDTA (pH = 8.0) for 3 min, and subsequently incubated with primary antibodies against MEX3A (Abcam, ab79046, 1:200) and PDE5A (Abcam, ab259945, 1:300) overnight at 4 °C. Following secondary antibody incubation, the slides were stained with DAB (YXBIOTECH, Guangzhou, China, #ZLI-9018). Jiangfeng scanner (KF-PRO-020, China) was used for image capturing. Evaluation of MEX3A or PDE5A staining was conducted independently by two pathologists. The H-score classification was performed as previously described.^49^

### Statistical analysis

All experiments were performed at least three times and the results are presented as mean ± standard deviation (S.D.). Statistical significance was determined by a two-tailed Student’s *t* test or a two-way ANOVA. The Chi-square test was used to analyze the significance of the correlation of MEX3A expression with clinicopathological features. Survival analysis was performed using the Kaplan-Meier curve and the Log-rank test. The correlation of MEX3A, circMPP6 and PDE5A expression in CRCs was analyzed using Pearson correlation analysis. Statistical significance was defined as *P* value < 0.05. All analyses were carried out using SPSS (version 25.0) or GraphPad Prism (version 8.0).

### Supplementary information

Supplementary_Materials

Movie S1

Movie S2

Movie S3

Movie S4

Movie S5

Movie S6

## Data Availability

The paper and Supplementary Materials contain all the necessary data for evaluating the conclusions. The MS data are accessible using PeptideAtlas Dataset ID: PASS03791. Flag-MEX3A RIP-Seq data have been submitted to the Sequence Read Archive (SRA) database under project PRJNA899006. MEX3A knockdown mRNA-Seq data have been submitted to the SRA database under project PRJNA899494.
