# Supplementary material for: Mex-3 RNA binding family member A (MEX3A)/circMPP6 complex promotes colorectal cancer progression by inhibiting autophagy
Source: Signal Transduct Target Ther. 2024 Apr 2;9:80. doi: 10.1038/s41392-024-01787-3 (PMC10987644; doi:10.1038/s41392-024-01787-3)

Supplementary Materials for

Mex-3 RNA binding family member A (MEX3A) /circMPP6 complex promotes colorectal cancer progression by inhibiting autophagy

Ri-Xin Chen^1,2,3#^, Shui-Dan Xu^2#^, Min-Hua Deng^2#^, Shi-Hui Hao^2#^, Jie-Wei Chen^2,4^, Xiao-Dan Ma^2^, Wei-Tao Zhuang^2^, Jing-Hua Cao^2^, Yong-Rui Lv^2^, Jin-Long Lin^2^, Si-Yu Li^2^, Gui-Bin Qiao^1^*, Dan Xie^2,4^*, Feng-Wei Wang^2^*

Correspondence to: wangfengw@sysucc.org.cn (Feng-Wei Wang) or

xiedan@sysucc.org.cn (Dan Xie) or

guibinqiao@126.com (Gui-Bin Qiao)

**This PDF file includes:**

Supplementary Materials and Methods

Figures. S1 to S16

Tables S1 to S4

Captions for Movies S1 to S6

**Other Supplementary Materials for this manuscript include the following:**

original and uncropped films of Western blotting

original Microscopy images

Movies S1 to S6

Supplementary Materials and Methods

Cell culture

The human kidney cell line 293T, and the colorectal cell lines DLD-1, SW480, and HCT116 were purchased from American Type Culture Collection (ATCC). All cells were cultured less than three months after resuscitation and were grown in a humidified incubator with 5% CO_2_ at 37 °C. DLD-1, SW480, and HCT116 cells were cultured in Roswell Park Memorial Institute 1640 (Invitrogen, California, USA, #C11875500BT), while 293T cells were cultured in Dulbecco’s Modified Eagle Medium (DMEM) (Invitrogen, California, USA, #C11995500BT). All the mediums were supplemented with 10% fetal bovine serum (FBS) (PAN, Aidenbach, Germany, #ST30-3302) and 1% Penicillin-Streptomycin solution (Biosharp, Hefei, China, #BL505A).

RNA extraction and Quantitative real-time PCR (qRT-PCR)

Total RNA was extracted using the TRIzol reagent (Invitrogen, California, USA, #15596018). The cDNA was generated using a Prime Script RT reagent Kit (Takara, Dalian, China, #RR036A or #RR037A). Quantitative real-time PCR was conducted using SYBR Green SuperMix (Bio-Rad, California, USA, #1725122). Either *U3* or *GAPDH* was used as an internal control. The primer sequences are listed in **Supplementary Table S4**.

Western blotting

Proteins were extracted using lysis buffer supplemented with a protease inhibitor cocktail (CWBIO, Beijing, China, #CW2200S), and quantified by a BCA Protein Assay kit (GLPBIO, Montclair, USA, #GK10009-2500T). Then the prepared protein lysates were resolved by 10% SDS-PAGE gels and transferred on a PVDF membrane (Roche, Basel, Switzerland, #3010040001). After blocking with 5% milk, the membranes were incubated with primary antibodies at 4 °C overnight and then hybridized with corresponding secondary antibodies at room temperature for 1 h. The signals were visualized by Chemistar™ High-sig ECL Western Blotting Substrate (Tanon, Shanghai, China, #180-5001W).

Plasmid construction and transfection

The MEX3A overexpression plasmid was obtained from Furuibio (Guangzhou, China), while the GST-tagged, EGFP-tagged, Flag-tagged MEX3A full-length or truncated plasmids, and circMPP6 WT or mutant plasmids were obtained from GeneCreate Biotech (Wuhan, China). The mRFP-GFP-LC3 plasmid was obtained from Addgene (MA, USA, #117413).

The plasmid psi-LVRU6GP was used for constructing the MEX3A, circMPP6, and PDE5A shRNAs and obtained from GeneCopoeia (MD, USA). The target sequences for constructing lentiviral shRNAs are listed in **Supplementary Table S4**.

Plasmid transfection was performed using jetPRIME (Polyplus Transfection, Strasbourg, France, #114-15). For stable transductions, lentivirus was packaged by co-transfecting the plasmids with the lentiviral packaging plasmids pMD2G, and psPAX2 (GeneCopoeia, MD, USA) into 293T cells. The supernatants containing viruses were collected at 48 h post-transfection. Puromycin or Geneticin was used to select stable cells.

Colony formation assay

Cells were seeded into 6-well plates at 500 cells per well and cultured with a complete medium for 10 days. After washing with phosphate-buffered saline (PBS) (Thermo Fisher Scientific, Waltham, USA, #C10010500BT), the cells were fixed with methanol for 30 min and stained with crystal violet (Beyotime, Shanghai, China, #C0121) for 20 min.

Transwell migration assay

Transwell chamber (BD Biosciences, New Jersey, USA, #353097) was placed into a 24-well plate filled with 500 μL medium of 30% FBS, and 2×10^5^ cells in 200 μL serum-free medium were added to the upper chamber. After incubation for 24 h (DLD-1), 36 h (HCT116), or 48 h (SW480), the migrated cells were fixed with methanol, stained with crystal violet, and then counted using a NIKON microscope (NIKON ECLIPSE 80i, Japan).

RNA-stability assay

Total RNA (2 μg) was incubated with 3 U/μg of RNase R (Lucigen, Madison, USA, # RNR07250) for 15 min at 37 °C. After treatment with RNase R, the expression levels of circMPP6 and *MPP6* mRNA were analyzed by qRT-PCR.

CRC cells were planted in 6-well plates. After 24 h, cells were treated with 5 μg/mL Actinomycin D and collected at the indicated time points. The expression levels of RNAs were analyzed by qRT-PCR.

Isolation of cytoplasmic and nuclear RNA

Cytoplasmic and nuclear RNA fractions were isolated by using PARIS™ Kit (Thermo Fisher Scientific, Waltham, USA, #AM1556). Cells were lysed in Cell Fraction Buffer on ice for 5 min. After centrifugation at 500× g for 3 min at 4 °C, the supernatant was collected as cytoplasmic fraction. The pellet was dissolved in Cell Disruption Buffer for nuclear isolation.

Antibodies

The following antibodies were used:

| Name | Manufacturer | Lot. No. | Source | Application |
| --- | --- | --- | --- | --- |
| MEX3A | Abcam | ab79046 | Rabbit | WB/IHC/IP /RIP/IF |
| LC3 | Proteintech | 14600-1-AP | Rabbit | WB |
| P62 | Proteintech | 18420-1-AP | Rabbit | WB |
| MOV10 | Proteintech | 10370-1-AP | Rabbit | WB/IF/RIP |
| PABP1 | Proteintech | 10970-1-AP | Rabbit | WB/IF |
| UPF1 | Abcam | ab109363 | Rabbit | WB/IF/RIP |
| PDE5A | Abcam | ab259945 | Rabbit | WB/IHC |
| PDE5A | Santa Cruz Biotechnology | sc-398747 | Mouse | IF |
| Flag-Tag | Cell Signal Technology | 14793S | Rabbit | WB/IP/RIP |
| GAPDH | Proteintech | 60004-1-Ig | Mouse | WB |

Figure. S1.

**
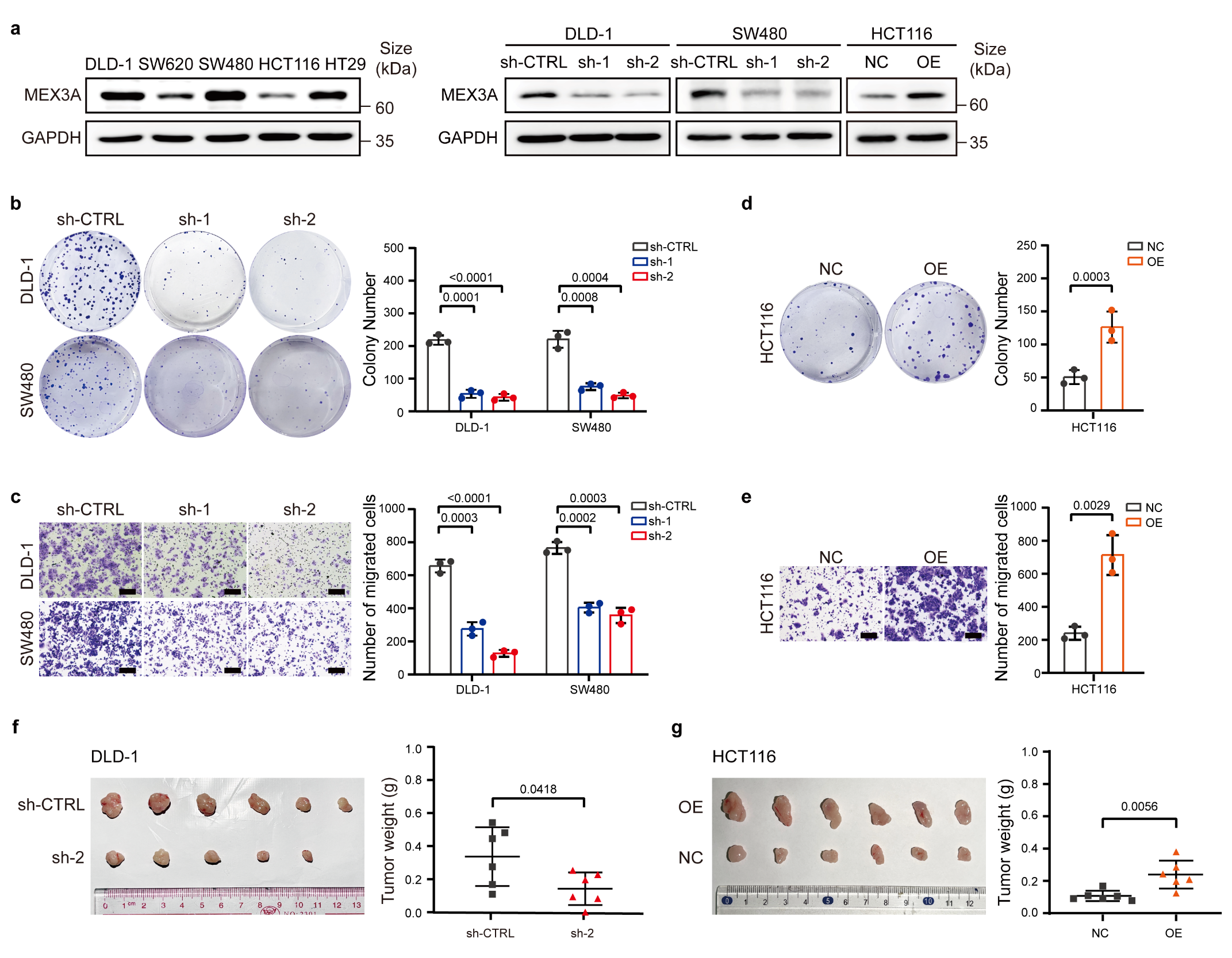
**

**Fig. S1** MEX3A accelerates the malignant properties of CRC cells. **a** Western blotting for MEX3A protein expression in indicated CRC cells. **b** Colony formation assay showing knockdown of MEX3A inhibited the cell growth of DLD-1 and SW480 cells. Left, representative images. Right, histograms of colony numbers. **c** Transwell migration assay showing knockdown of MEX3A inhibited the migration abilities of DLD-1 and SW480 cells. Left, representative images. Scale bar, 200 μm. Right, histograms of migrated cell numbers. **d** Colony formation assay showing overexpression of MEX3A promoted the cell growth of HCT116 cells. Left, representative images. Right, histograms of colony numbers. **e** Transwell migration assay showing overexpression of MEX3A promoted the migration abilities of HCT116 cells. Left, representative images. Scale bar, 200 μm. Right, histograms of migrated cell numbers. **f-g** The subcutaneous xenograft model showing knockdown (**f**) or overexpression (**g**) of MEX3A inhibited or promoted subcutaneous tumor formation in BALB/c nude mice (*n* = 6). Left, subcutaneous tumor. Right, histograms of subcutaneous tumor weights. Data are represented as mean ± S.D. from three independent experiments (**b**, **c**, **d**, **e**). The *P* value was determined by a two-tailed unpaired Student’s *t* test (**b**, **c**, **d**, **e**, **f**, **g**).

Figure. S2.

**
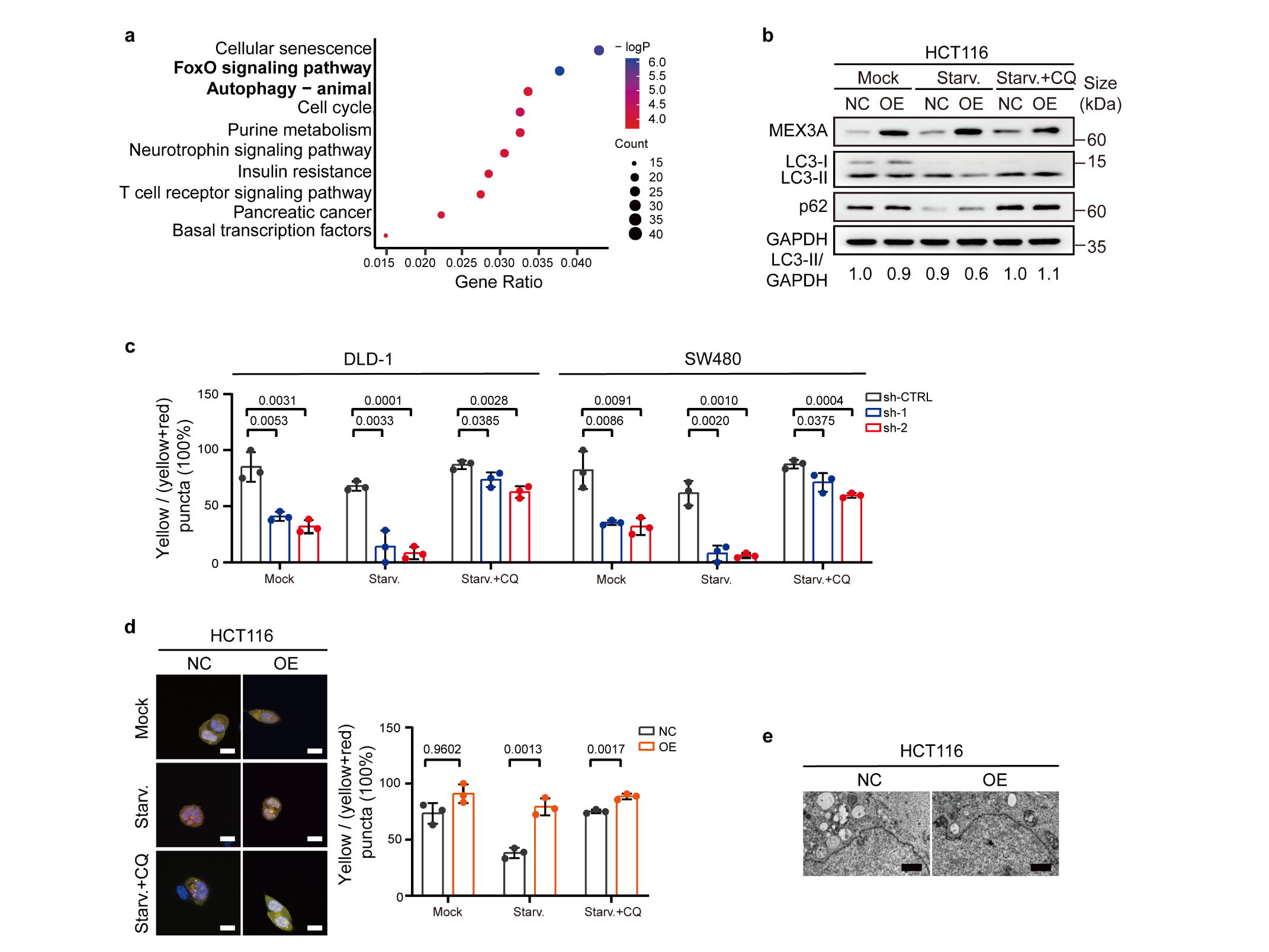
**

**Fig. S2** MEX3A suppresses autophagy of CRC cells. **a** KEGG pathway enrichment analysis showing the mRNAs binding to MEX3A involved in autophagy-related pathways. The size of the dot indicates the number of genes per pathway, and the color indicates the *P* value. **b** Western blotting for LC3 conversion and p62 expression in control (NC) or MEX3A-overexpression (OE) cells with or without serum or CQ. GAPDH was used as an internal control. **c** The quantification of the yellow (mRFP+/GFP+) puncta in indicated cells in mRFP-GFP-LC3 assay in Fig. 1h. **d** Detection of autophagic flux with the mRFP-GFP-LC3 reporter in indicated cells. Left, representative images. Scale bar, 10 μm. Right, the quantification of the yellow (mRFP+/GFP+) puncta in indicated cells in mRFP-GFP-LC3 assay. **e** Representative TEM images of the autophagosomes and/or autolysosomes in control (NC) or MEX3A-overexpression (OE) cells. Scale bar, 500 nm. Data are represented as mean ± S.D. from three independent experiments (**c**, **d**). The *P* value was determined by a two-tailed unpaired Student’s *t* test (**c**, **d**).

Figure. S3.


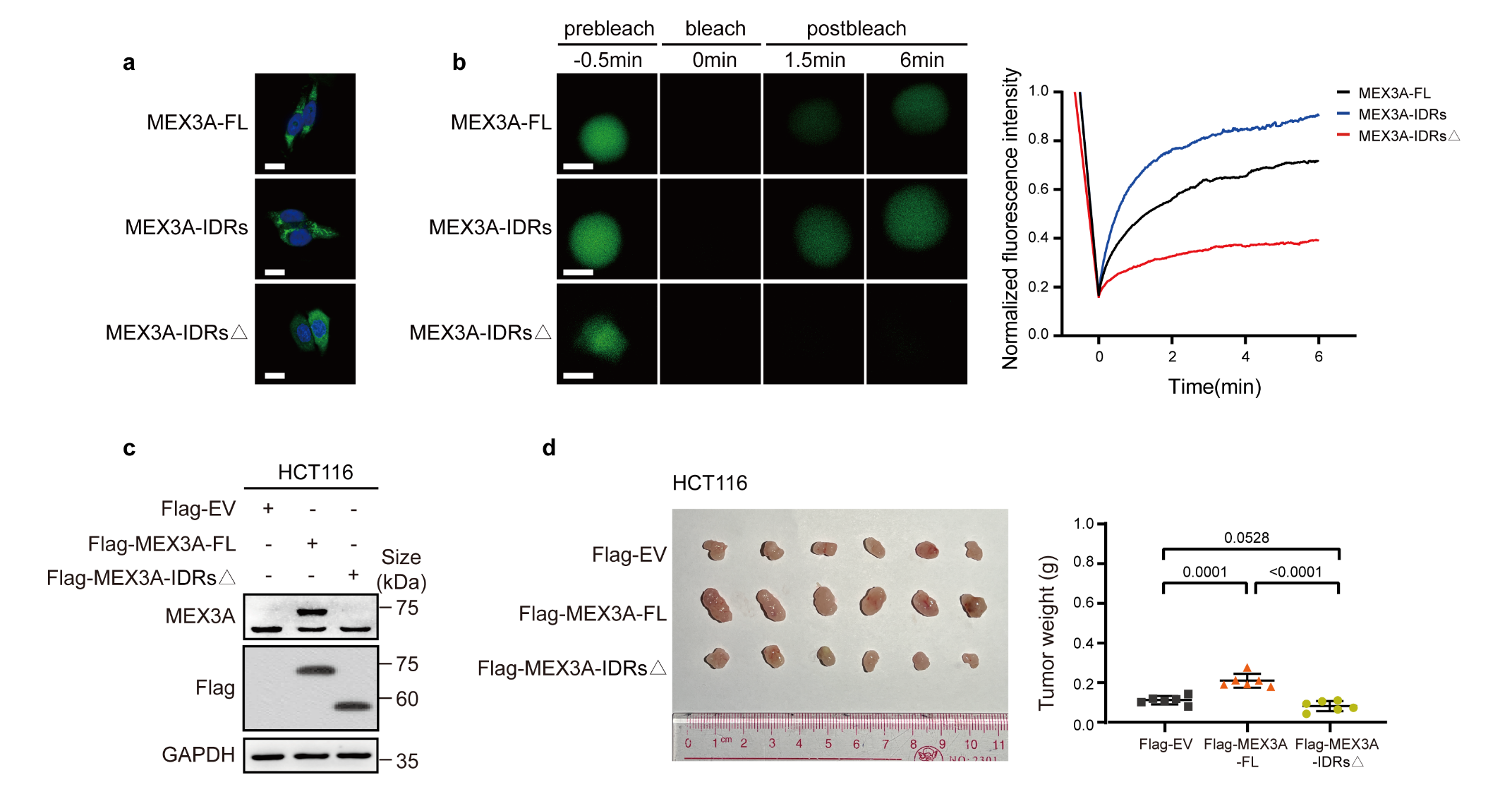


**Fig. S3** IDRs of MEX3A promote CRC tumorigenesis. **a** Immunofluorescence (IF) assay showing EGFP-MEX3A-FL, EGFP-MEX3A-IDRs, EGFP-MEX3A-IDRs△(green) formed cytoplasmic puncta in SW480 cells. Nuclei were stained with DAPI (blue). Scale bar, 10 μm. **b** Fluorescence recovery after photobleaching (FRAP) assay of the droplets formed by EGFP-MEX3A-FL and EGFP-MEX3A-IDRs in SW480 cells. Left, representative images. Scale bar, 500 nm. Right, quantification of fluorescence intensity recovery after photobleaching. **c** Western blotting for MEX3A protein expression in HCT116 indicated cells. **d** The subcutaneous xenograft model showing overexpression of MEX3A-IDRs△ attenuated tumor formation in BALB/c nude mice compared to MEX3A-FL (*n* = 6). Left, subcutaneous tumor. Right, histograms of subcutaneous tumor weights. The *P* value was determined by a two-tailed unpaired Student’s *t* test (**d**).

Figure. S4.


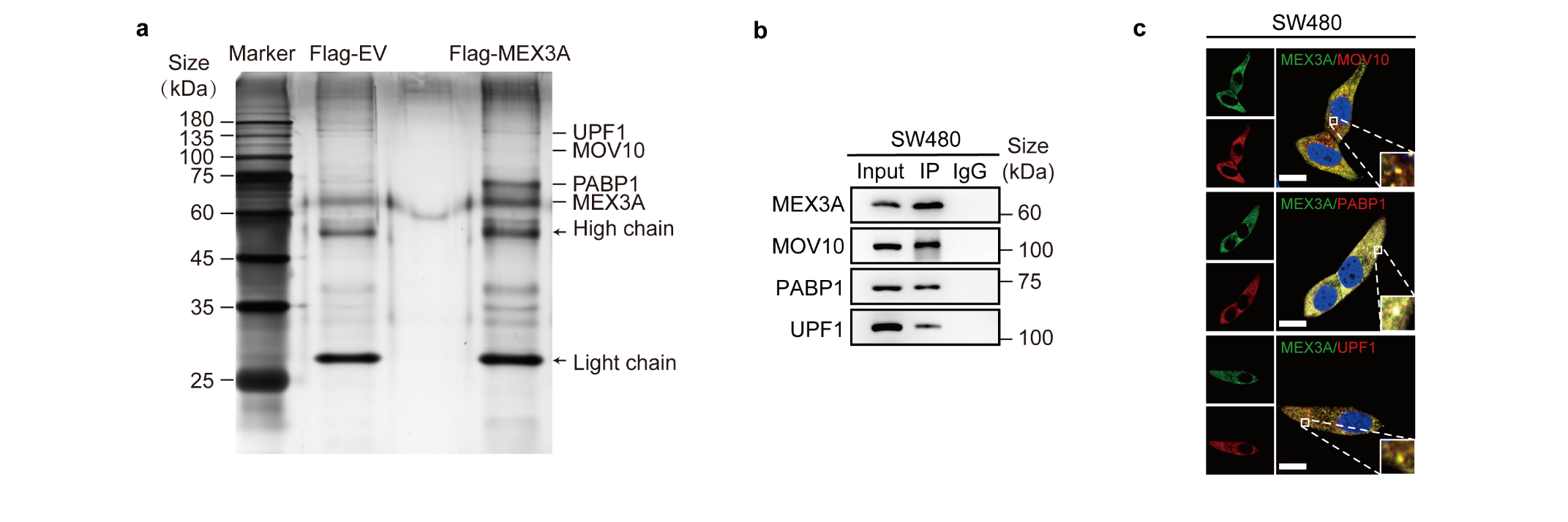


**Fig. S4** MEX3A physically interacts with PBs components. **a** Silver staining of the protein enriched by anti-Flag antibody in Flag-MEX3A-overexpression 293T cells. **b** Western blotting of MEX3A-IP assay showing the binding between MEX3A and the PBs proteins in SW480 cells. **c** IF assay showing that MEX3A was colocalized with the PBs proteins MOV10, PABP1, and UPF1 in SW480 cells. Nuclei were stained with DAPI (blue). Scale bar, 10 μm.

Figure. S5.


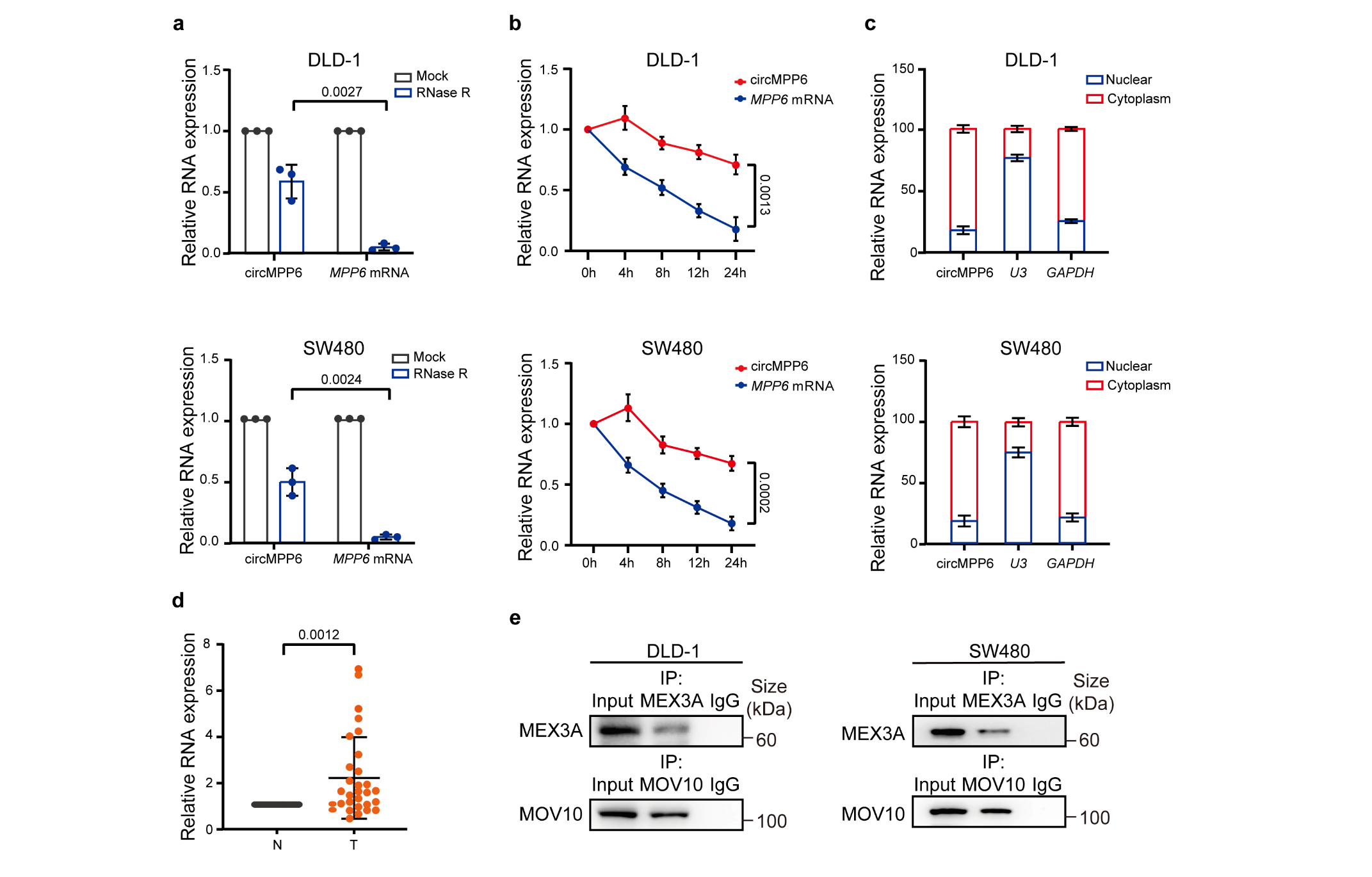


**Fig. S5** MEX3A forms a complex with circMPP6 in PBs. **a** qRT-PCR analysis for circMPP6 and *MPP6* mRNA expression after treatment with RNase R in DLD-1 and SW480 cells. **b** qRT–PCR analysis for the expression of circMPP6 and *MPP6* mRNAs after treatment with Actinomycin D at the indicated time points in DLD-1 and SW480 cells. **c** Isolation of cytoplasmic and nuclear RNA showing that circMPP6 was predominantly localized in the cytoplasm. *U3* and *GAPDH* were applied as positive controls in the nucleus and cytoplasm. **d** qRT-PCR analysis for the expression of circMPP6 in 30 CRC tissues (T) and matched adjacent normal tissues (N). Data are represented as mean ± S.D., the *P* value was determined by a two-tailed paired Student’s *t* test. **e** Western blotting showing the RIP efficiency of anti-MEX3A and anti-MOV10 antibodies in CRC cells. Data are represented as mean ± S.D. from three independent experiments (**a, b, c**). The *P* value was determined by a two-tailed unpaired Student’s *t* test (**a**) or a two-way ANOVA (**b**).

Figure. S6.


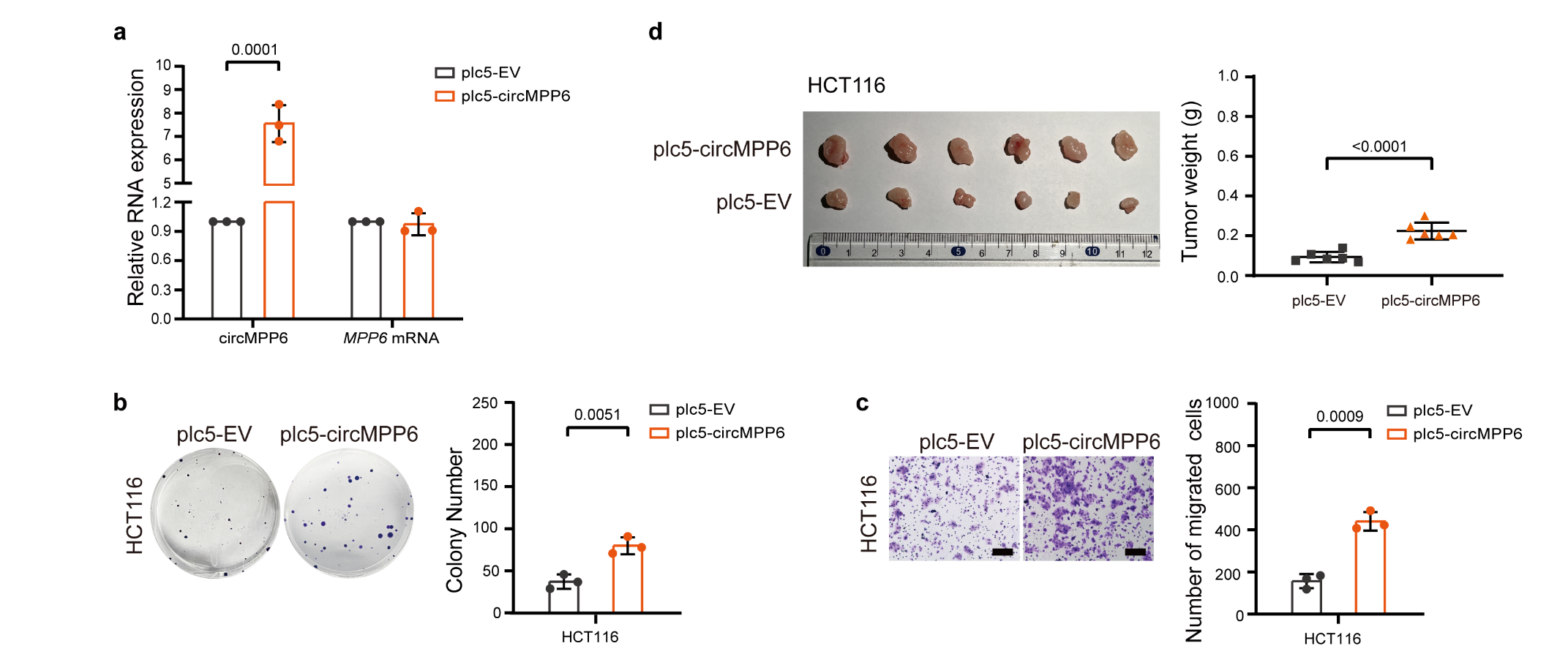


**Fig. S6** CircMPP6 modulates CRC malignant properties. **a** qRT-PCR for circMPP6 and *MPP6* mRNA in CRC cells transfected with control vector or circMPP6 overexpression plasmid. **b** Colony formation assay showing overexpression of circMPP6 promoted the cell growth of HCT116 cells. Left, representative images. Right, histograms of colony numbers. **c** Transwell migration assay showing overexpression of circMPP6 promoted the migration abilities of HCT116 cells. Left, representative images. Scale bar, 200 μm. Right, histograms of migrated cell numbers. **d** The subcutaneous xenograft model showing overexpression of circMPP6 promoted subcutaneous tumor formation in BALB/c nude mice (*n* = 6). Left, subcutaneous tumor. Right, histograms of subcutaneous tumor weights. Data are represented as mean ± S.D. from three independent experiments (**a**, **b**, **c**). The *P* value was determined by a two-tailed unpaired Student’s *t* test (**a**, **b**, **c**, **d**).

Figure. S7.

**
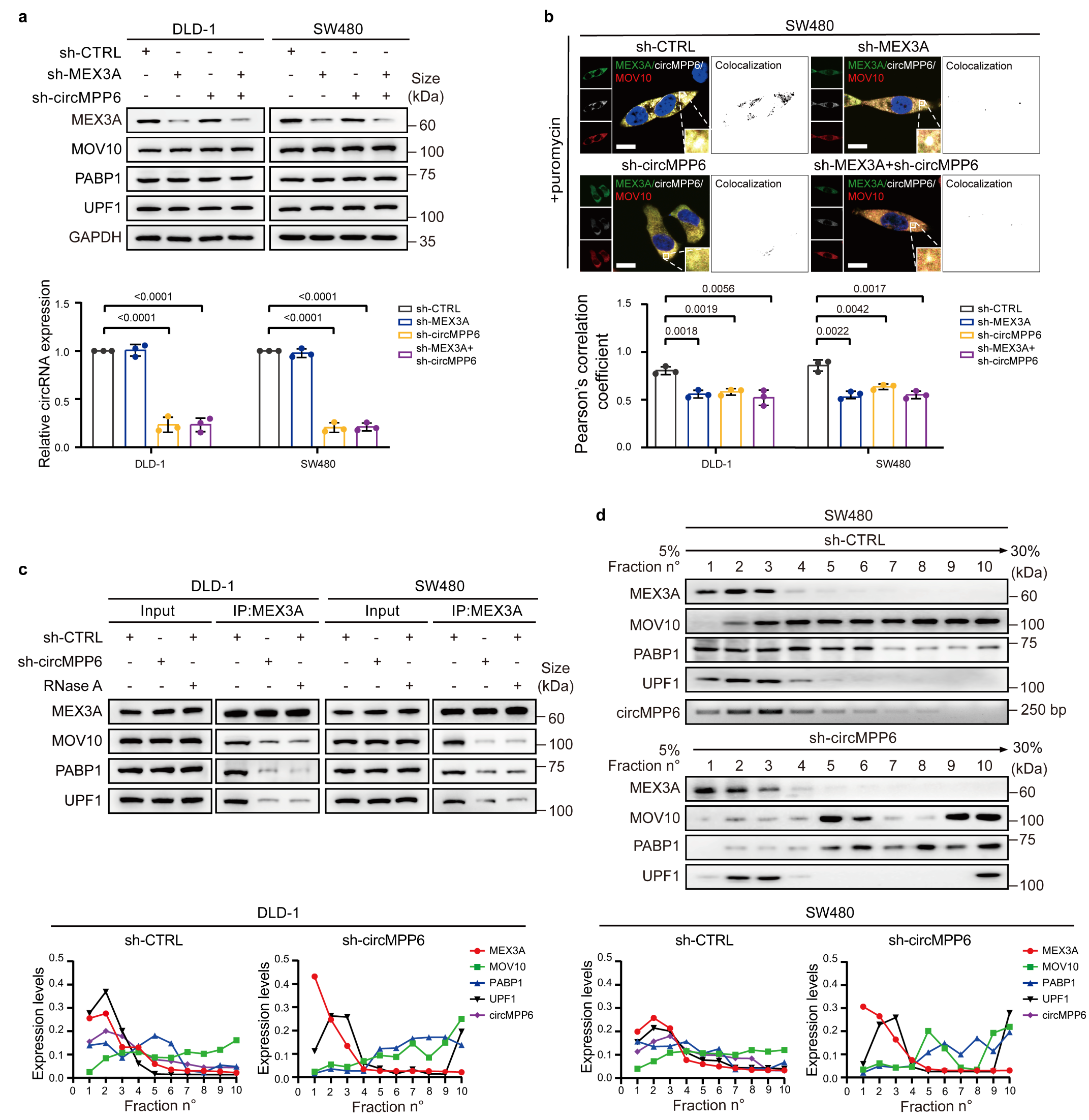
**

**Fig. S7** CircMPP6 facilitates PBs aggregation by strengthening MEX3A-PBs components interaction. **a** Top, Western blotting for the protein expression of MEX3A and PBs proteins upon MEX3A and/or circMPP6 knockdown. GAPDH was used as the loading control. Bottom, qRT-PCR for the expression of circMPP6 upon MEX3A and/or circMPP6 knockdown. **b** IF-FISH assays showing that the colocalization of MEX3A/circMPP6/MOV10 was decreased upon MEX3A and/or circMPP6 knockdown after treatment with puromycin in SW480 cells. Top, representative images. Nuclei were stained with DAPI (blue). Scale bar, 10 µm. Bottom, Pearson’s correlation coefficient analysis. **c** Western blotting of MEX3A-IP assay showing the association of MEX3A with other PBs proteins upon circMPP6 knockdown or RNase A treatment. **d** Top, sucrose gradient fraction analysis of a whole-cell lysate from control and circMPP6-knockdown SW480 cells. Bottom, protein levels quantified by Image J and circRNA level examined by qRT-PCR in cell lysates of control and circMPP6-knockdown cells. Data are represented as mean ± S.D. from three independent experiments (**a**, **b**). The *P* value was determined by a two-tailed unpaired Student’s *t* test (**a**, **b**).

Figure. S8.


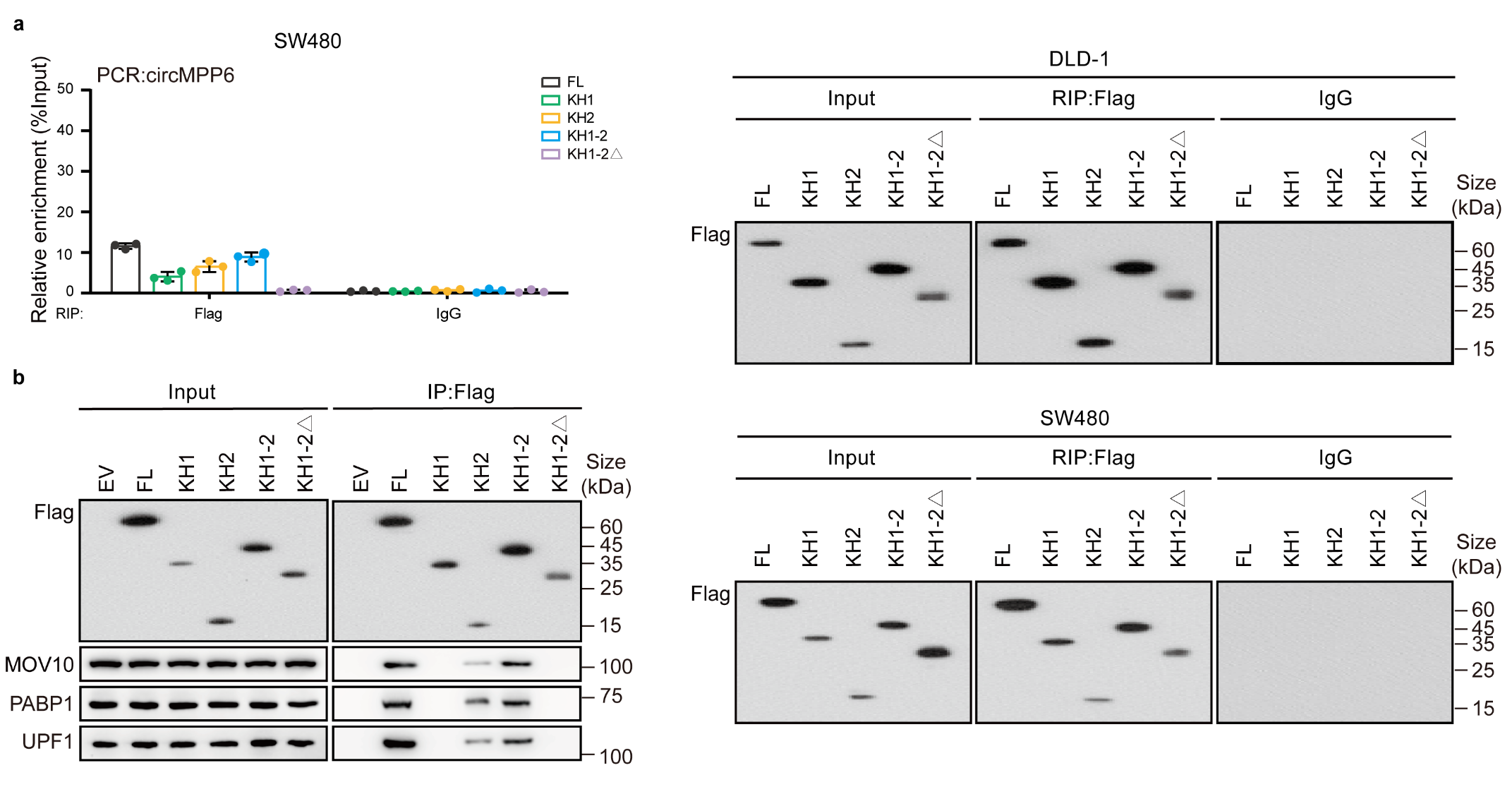


**Fig. S8** The KH2 domain of MEX3A bound with circMPP6 is required for recruiting PBs components. **a** Left, qRT–PCR analysis of RIP assay in SW480 showing the interactions between circMPP6 and each truncation. Right, Western blotting of RIP assay in CRC cells transfected with plasmids encoding Flag-tagged full-length or truncated MEX3As. **b** Western blotting of Flag-IP assay in SW480 cells showing the interactions between PBs proteins and each truncation. Data are represented as mean ± S.D. from three independent experiments (**a**).

Figure. S9.


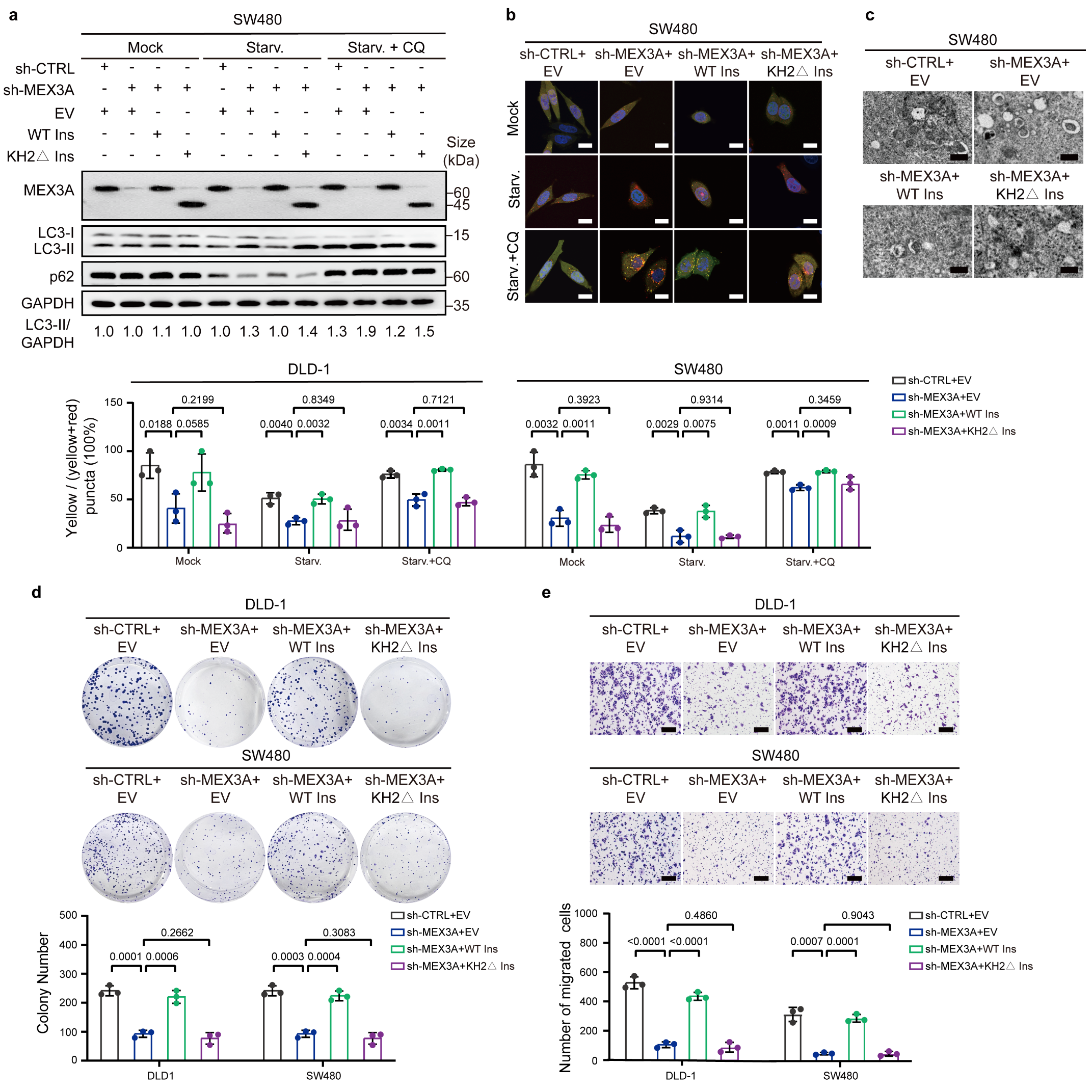


**Fig. S9** The KH2 domain of MEX3A bound with circMPP6 plays an important role in CRC cell progression. **a** Western blotting showing the changes of LC3 conversion and p62 expression upon MEX3A knockdown could be rescued by overexpression of WT, but not the mutant MEX3A in SW480 cells. **b**-**c** Representative images of the autophagic flux with the mRFP-GFP-LC3 reporter (**b**), and ultrastructural autophagosomes and/or autolysosomes (**c**) showing that MEX3A knockdown-induced autophagy could be counteracted by overexpression of WT, but not the mutant MEX3A in SW480 cells. Scale bar, 10 μm (**b**), 500 nm (**c**). **d** Colony formation assay showing that the inhibited cell growth could be recovered by overexpression of WT, but not the mutant MEX3A in CRC cells. **e** Transwell migration assay showing that the inhibited cell migration could be restored by overexpression of WT, but not the mutant MEX3A in CRC cells. Scale bar, 200 μm. Data are represented as mean ± S.D. from three independent experiments (**b**, **d**, **e**). The *P* value was determined by a two-tailed unpaired Student’s *t* test (**b**, **d**, **e**).

Figure. S10.


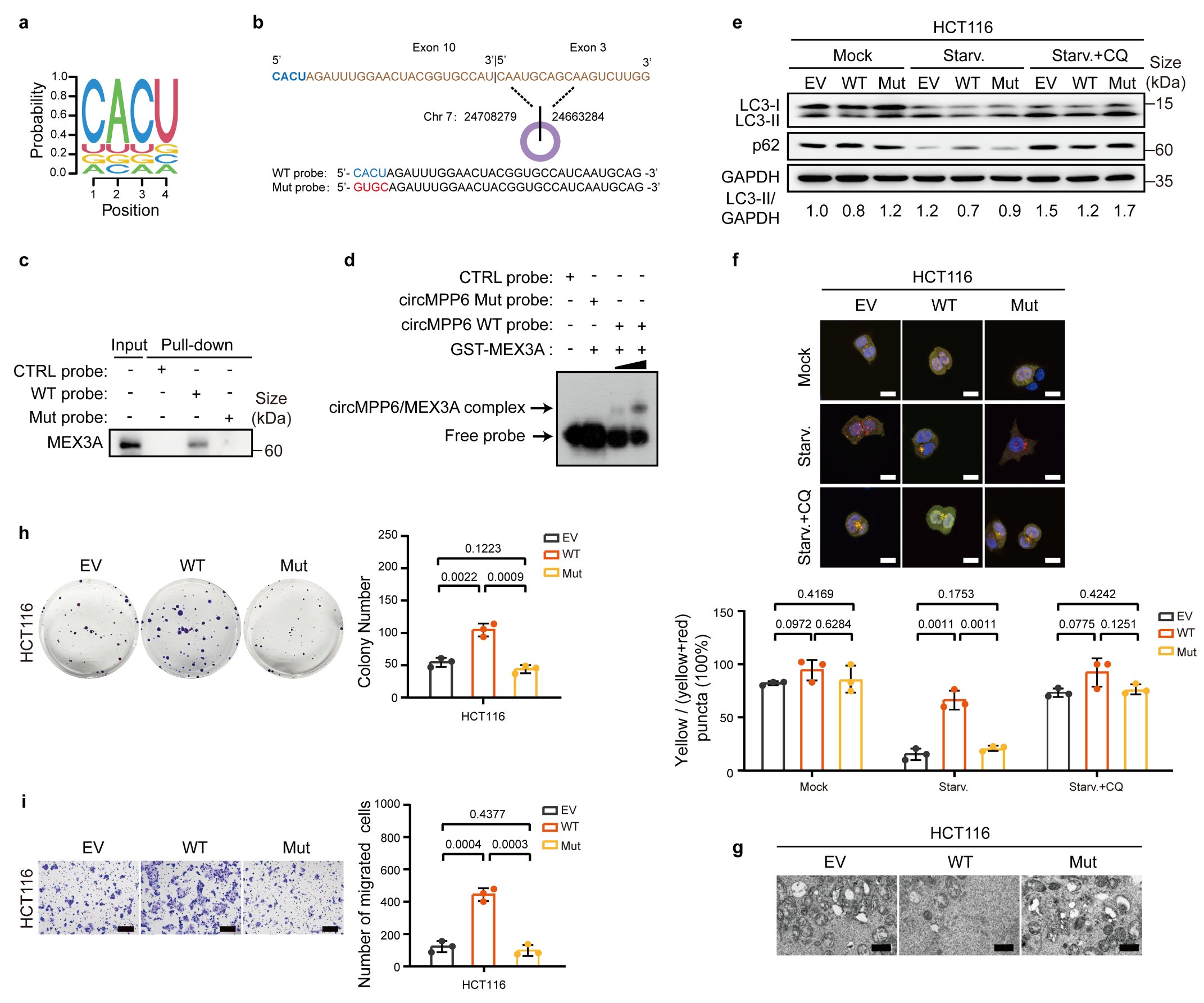


**Fig. S10** The CACU motif of circMPP6 bound with MEX3A plays an important role in CRC cell progression. **a** CACU RNA binding motif analysis from Flag-MEX3A RIP-seq. **b** Top, schematic illustration showing the CACU motif located at exon 10-exon 3 junction region of circMPP6. Bottom, the sequence of RNA probe for RNA Pull-down and RNA-EMSA assays. **c** Western blotting of RNA Pull-down assay showing the specific association of MEX3A with biotin-labeled oligonucleotides containing CACU motif from circMPP6. **d** RNA-EMSA assay showing the binding ability of purified MEX3A with biotin-labeled oligonucleotides containing CACU motif from circMPP6. **e** Western blotting showing the changes of LC3 conversion and p62 expression upon overexpression of circMPP6 WT or Mut in HCT116 cells. **f**-**g** Representative images of the autophagic flux with the mRFP-GFP-LC3 reporter (**f**), and ultrastructural autophagosomes and/or autolysosomes (**g**) showing that overexpression of circMPP6 Mut enhanced HCT116 cells autophagy compared to circMPP6 WT. Scale bar, 10 μm (**f**), 500 nm (**g**). **h** Colony formation assay showing that overexpression of circMPP6 Mut attenuated HCT116 cells growth compared to circMPP6 WT. **i** Transwell migration assay showing that overexpression of circMPP6 Mut attenuated HCT116 cells migration compared to circMPP6 WT. Scale bar, 200 μm. Data are represented as mean ± S.D. from three independent experiments (**f**, **h**, **i**), the *P* value was determined by a two-tailed unpaired Student’s *t* test (**f**, **h**, **i**).

Figure. S11.

**
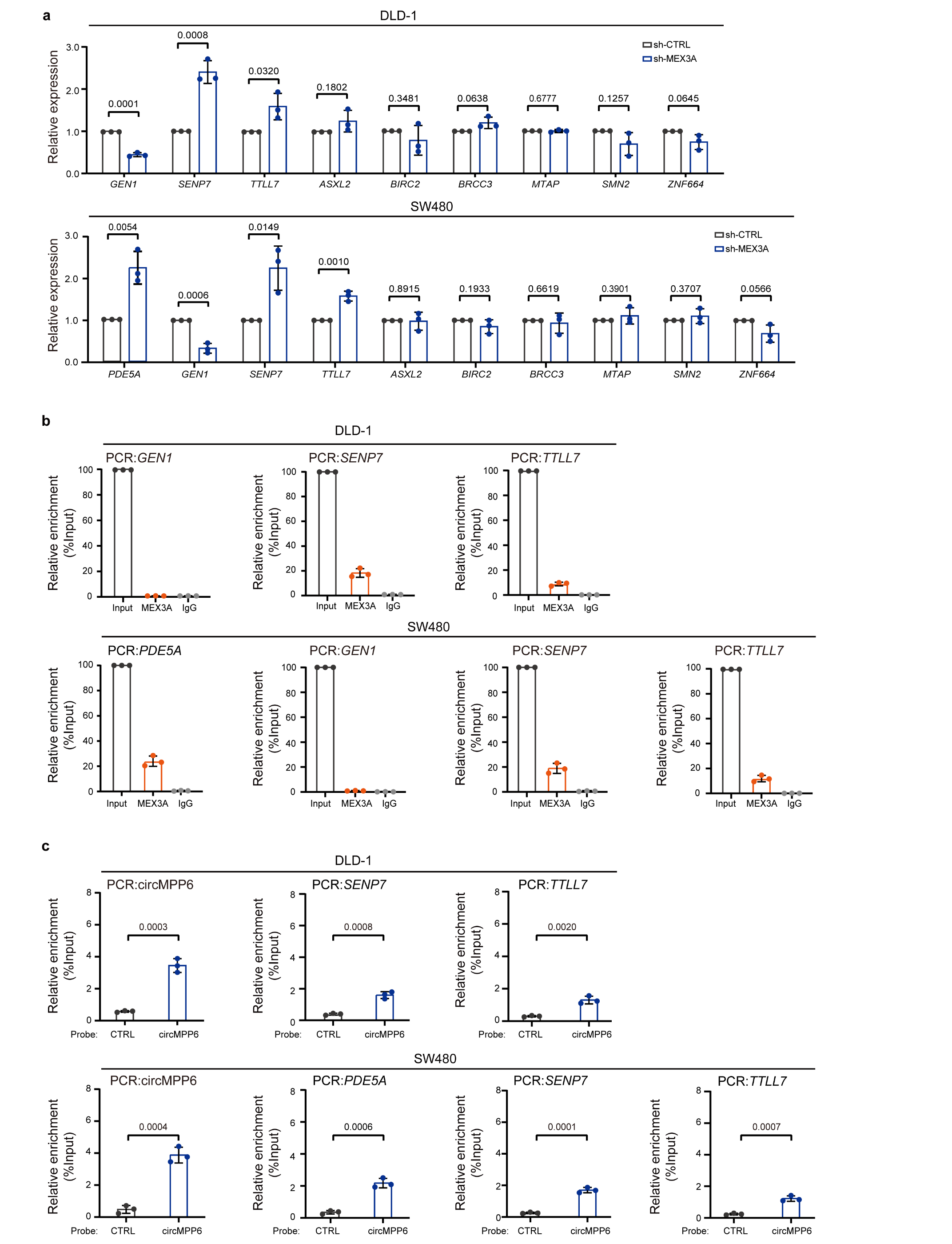
**

**Fig. S11** The MEX3A/circMPP6 complex modulates several mRNAs. **a** qRT–PCR analysis for the mRNA expression of the downstream targets which were selected in Fig. 5a in MEX3A-knockdown CRC cells. **b** qRT–PCR analysis of RIP assay showing the enrichment of *GEN1*, *SENP7*, *TTLL7*, and *PDE5A* mRNA associated with MEX3A protein in CRC cells. IgG antibody served as the negative control. **c** qRT-PCR analysis verifying the circMPP6 Pull-down efficiency and the interaction of *PDE5A* mRNA and other candidates with circMPP6 in CRC cells. Data are represented as mean ± S.D. from three independent experiments (**a**, **b**, **c**), the *P* value was determined by a two-tailed unpaired Student’s *t* test (**a**, **c**).

Figure. S12.

**
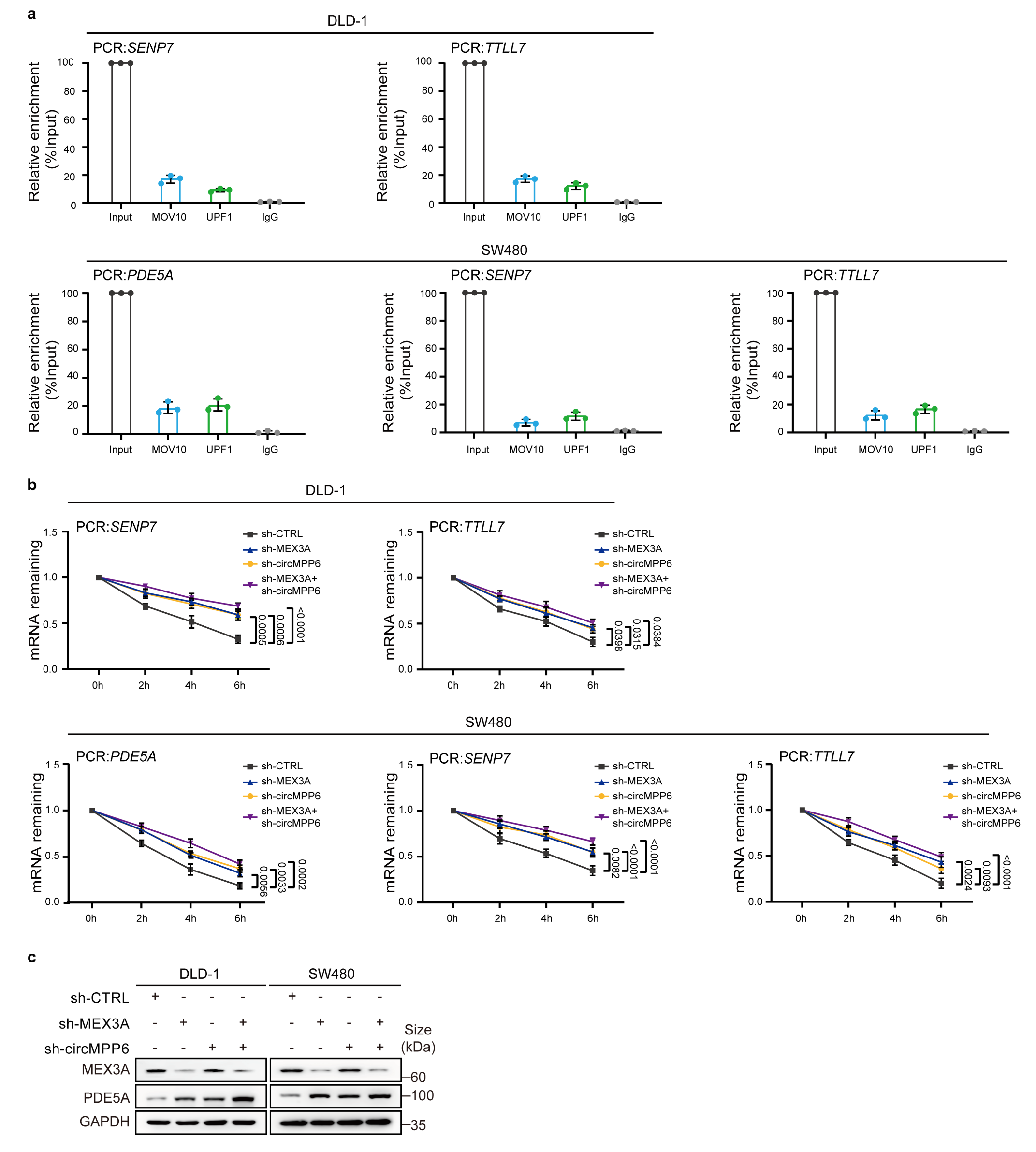
**

**Fig. S12** The MEX3A/circMPP6 complex mediated PBs aggregation affects mRNA decay. **a** qRT–PCR analysis of RIP analysis verifying the binding between mRNA candidates with MOV10 or UPF1 in DLD-1 and SW480 cells. **b** qRT-PCR analysis for the expression of mRNA candidates after treatment with Actinomycin D at the indicated time points upon MEX3A and/or circMPP6 knockdown in DLD-1 and SW480 cells. **c** Western blotting showing the expression of PDE5A protein upon MEX3A and /or circMPP6 knockdown. Data are represented as mean ± S.D. from three independent experiments (**a**, **b**), the *P* value was determined by a two-way ANOVA (**b**).

Figure. S13.


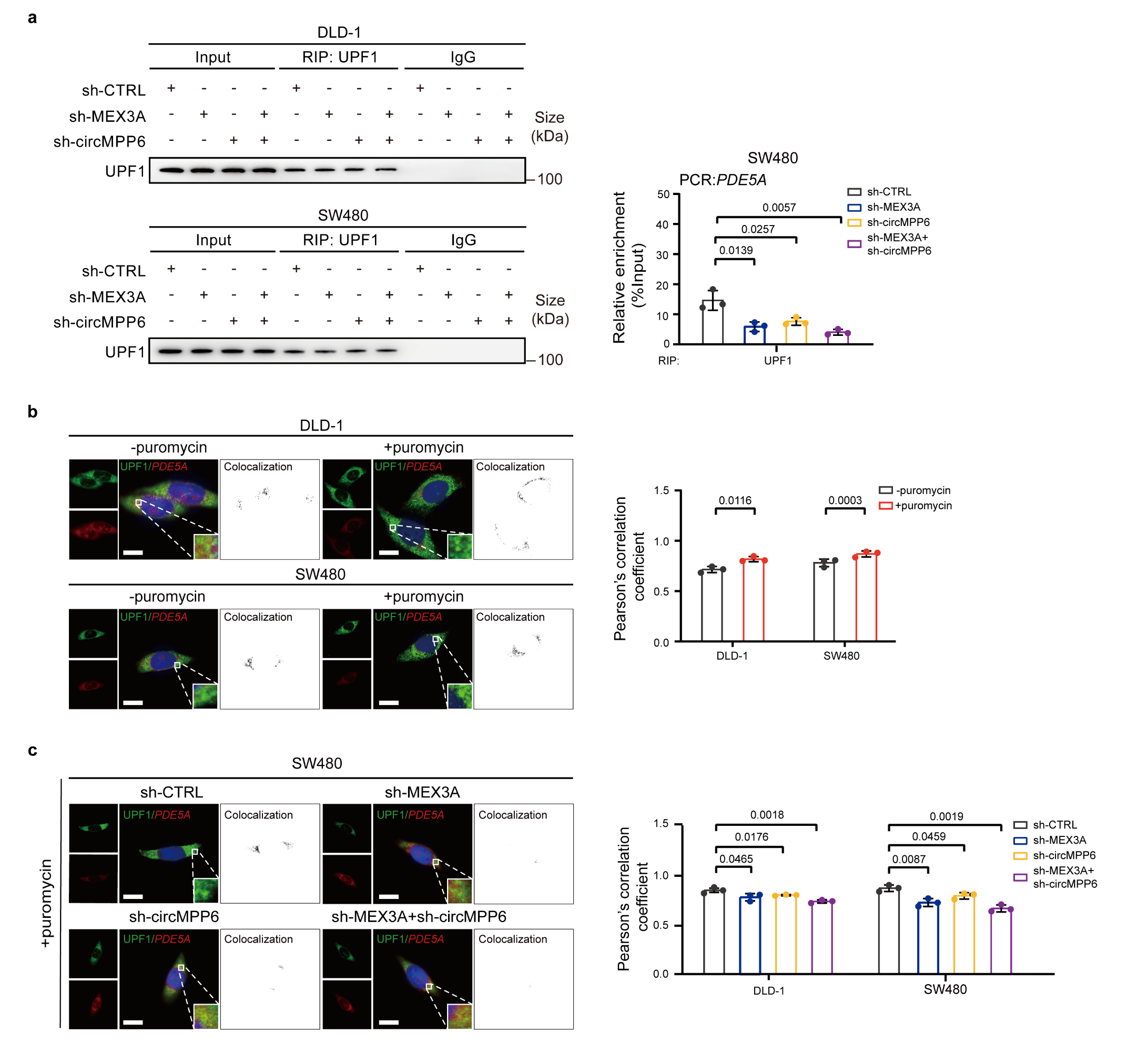


**Fig. S13** The MEX3A/circMPP6 complex promotes the interactions between UPF1 and *PDE5A* mRNA to trigger mRNA decay. **a** Left, Western blotting showing the RIP efficiency of anti-UPF1 antibody. Right, qRT–PCR analysis of RIP assay showing the association of *PDE5A* mRNA with UPF1 upon MEX3A and/or circMPP6 knockdown in SW480 cells. **b** IF-FISH assays showing the colocalization of UPF1/*PDE5A* complex and the fluorescence intensity of *PDE5A* mRNA with or without puromycin. Left, representative images. Nuclei were stained with DAPI (blue). Scale bar, 10 µm. Right, Pearson’s correlation coefficient analysis. **c** IF-FISH assays showing the colocalization of the UPF1/*PDE5A* complex upon MEX3A and/or circMPP6 knockdown after treatment with puromycin in SW480 cells. Left, representative images. Nuclei were stained with DAPI (blue). Scale bar, 10 µm. Right, Pearson’s correlation coefficient analysis. Data are represented as mean ± S.D. from three independent experiments (**a**, **b**, **c**), the *P* value was determined by a two-tailed unpaired Student’s *t* test (**a**, **b**, **c**).

Figure. S14.


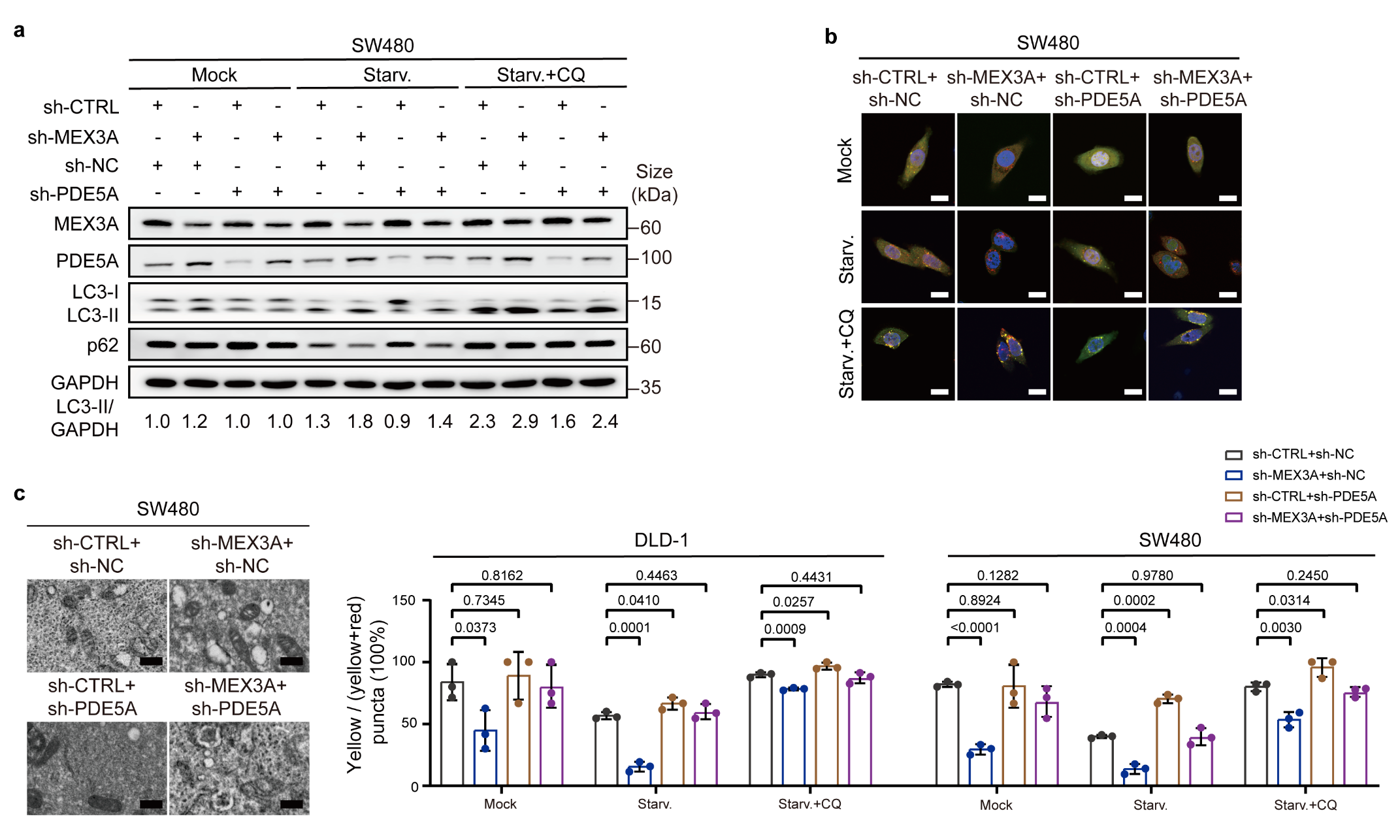


**Fig. S14** MEX3A modulates CRC autophagy through the PDE5A pathway. **a** Western blotting showing the changes of LC3 conversion and p62 expression upon MEX3A knockdown could be rescued by PDE5A silencing in SW480 cells. **b**-**c** Representative images of the autophagic flux with the mRFP-GFP-LC3 reporter (**b**), and ultrastructural autophagosomes and/or autolysosomes (**c**) showing that MEX3A knockdown-induced autophagy could be counteracted by PDE5A silencing in SW480 cells. Scale bar, 10 μm (**b**), 500 nm (**c**). Data are represented as mean ± S.D. from three independent experiments (**b**), the *P* value was determined by a two-tailed unpaired Student’s *t* test (**b**).

Figure. S15.

**
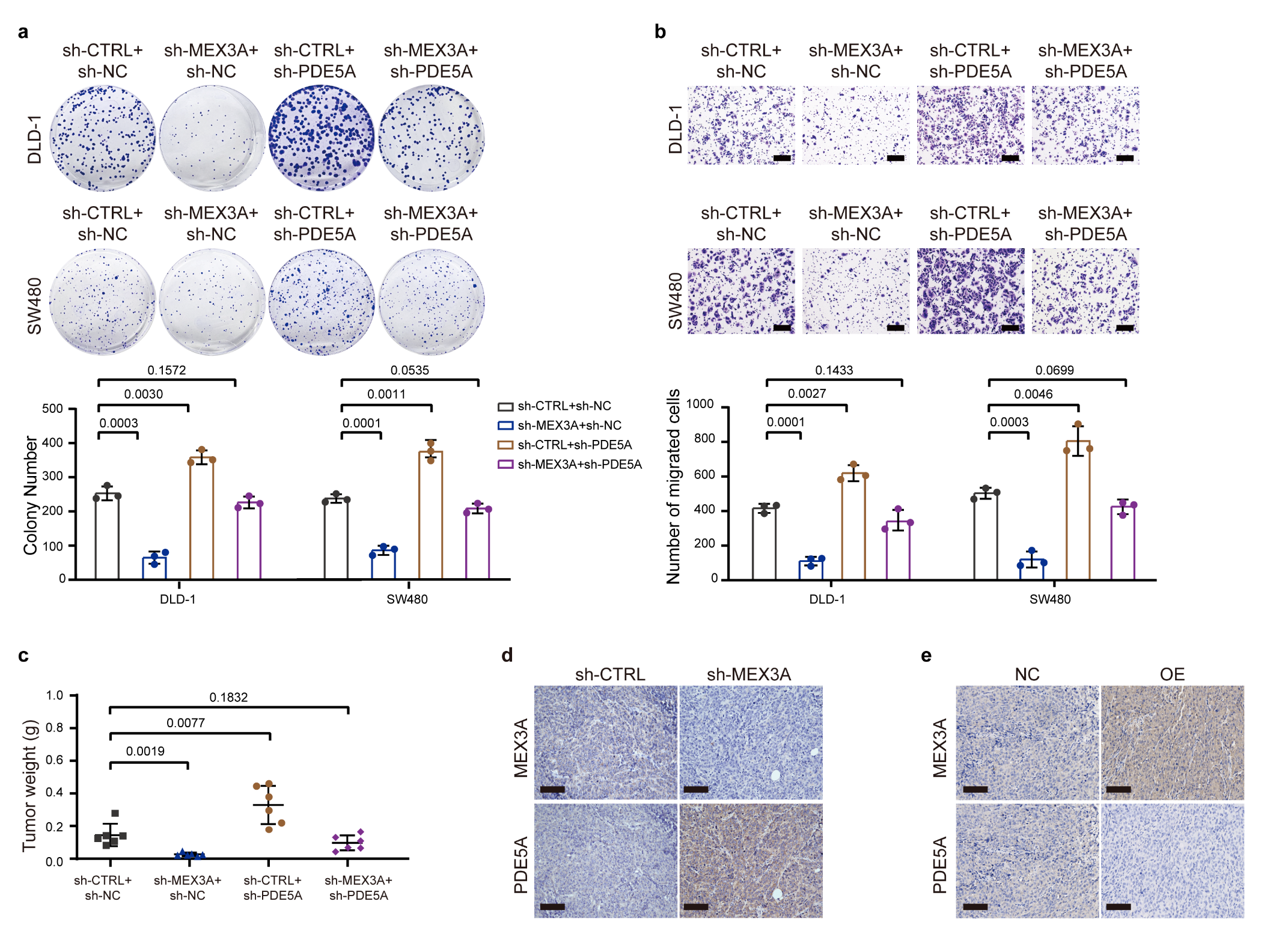
**

**Fig. S15** MEX3A modulates CRC malignant properties through the PDE5A pathway. **a** Colony formation assay showing that decreased cell growth in MEX3A-knockdown CRC cells was rescued by PDE5A silencing. Top, representative images. Bottom, histograms of colony numbers. **b** Transwell migration assay showing decreased cell migration abilities in MEX3A-knockdown CRC cells were rescued by PDE5A silencing. Top, representative images. Scale bar, 200 μm. Bottom, histograms of migrated cell numbers. **c** The histograms of subcutaneous tumor weights in Fig. 5l. **d** IHC staining assay of transplanted tumors of nude mice with injection of MEX3A-knockdown CRC cells showing increased expression of PDE5A. Scale bar, 100 μm. **e** IHC staining assay of transplanted tumors of nude mice with the injection of MEX3A-overexpression CRC cells showing decreased expression of PDE5A. Scale bar, 100 μm. Data are represented as mean ± S.D. from three independent experiments (**a**, **b**), the *P* value was determined by a two-tailed unpaired Student’s *t* test (**a**, **b**, **c**).

Figure. S16.

**
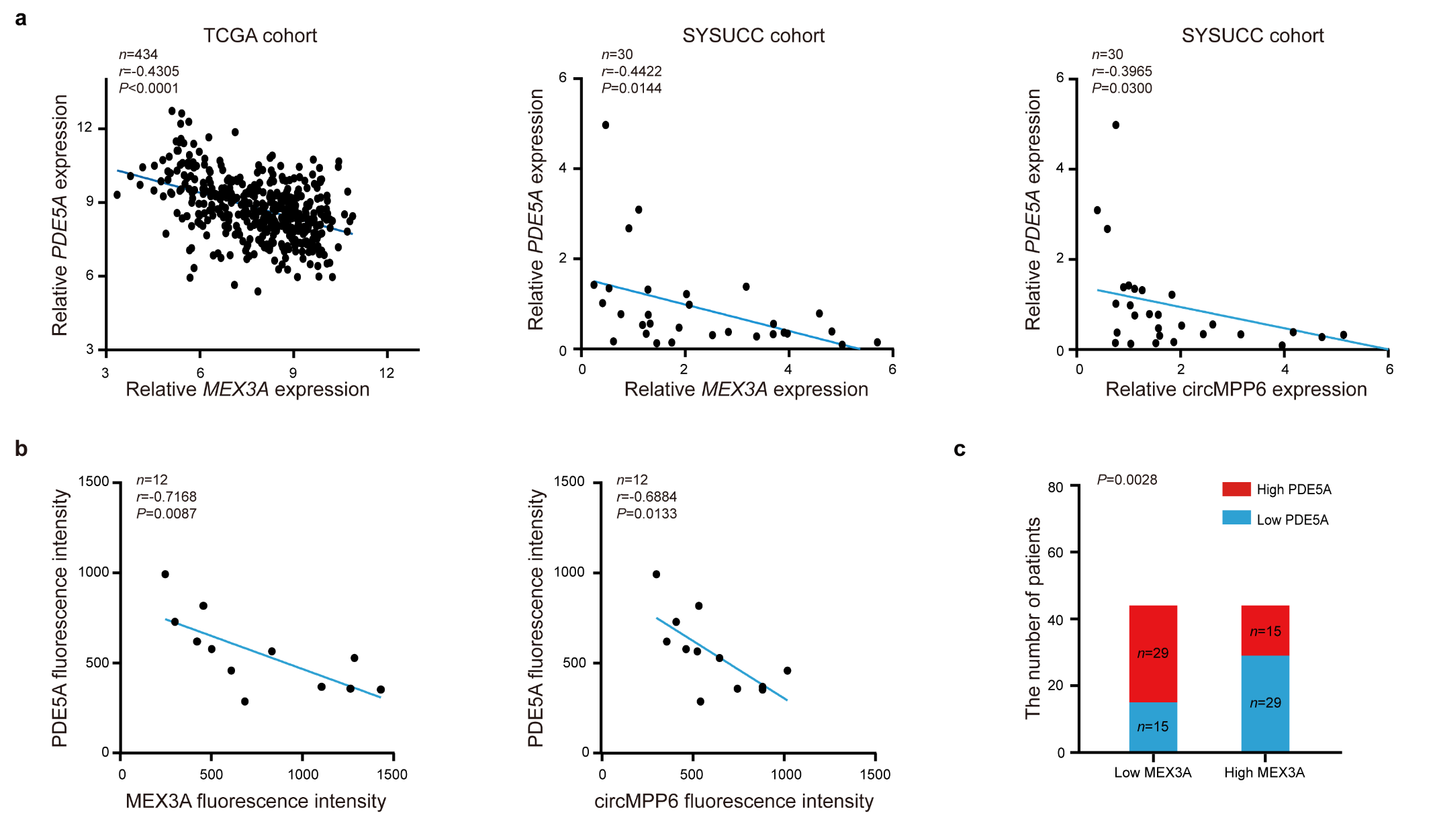
**

**Fig. S16** MEX3A/circMPP6-PDE5A axis is clinically correlated with CRC outcomes. **a** Left, middle, correlation of *MEX3A* mRNA abundance with *PDE5A* mRNA expression in the TCGA CRC cohort (left) and the SYSUCC cohort (middle). Right, correlation of circMPP6 RNA abundance with *PDE5A* mRNA expression in the SYSUCC cohort. The *P* values were determined by Pearson correlation analysis. **b** RNA-FISH and double immunofluorescence assays showing a negative correlation between the expression levels of PDE5A and those of MEX3A and circMPP6 in 12 CRCs. The *P* values were determined by Pearson correlation analysis. **c** Statistical analysis of the MEX3A and PDE5A protein expression verified by IHC. The *P* value was determined by a Chi-square test.

Table S1.

| **Correlation between MEX3A expression and clinicopathological parameters of CRC patients** | | | | |
| --- | --- | --- | --- | --- |
| Variable | Number of cases | MEX3A expression | | *P** |
|  |  | Low expression | High expression |  |
| Age (Year) |  | 44 | 44 | 0.669 |
| < 60 | 48 | 23 | 25 |  |
| ≥ 60 | 40 | 21 | 19 |  |
| Gender |  |  |  | 0.514 |
| Male | 53 | 25 | 28 |  |
| Female | 35 | 19 | 16 |  |
| Tumor location |  |  |  | 0.291 |
| Rectum | 18 | 11 | 7 |  |
| Colon | 70 | 33 | 37 |  |
| Histological grade (WHO) | |  |  | 0.580 |
| G1-2 | 16 | 7 | 9 |  |
| G3 | 72 | 37 | 35 |  |
| Clinical stage |  |  |  | 0.009 |
| I+II | 52 | 32 | 20 |  |
| III+IV | 36 | 12 | 24 |  |
| pT status |  |  |  | 0.001 |
| T1-T2 | 27 | 21 | 6 |  |
| T3-T4 | 61 | 23 | 38 |  |
| Lymph node status | |  |  | 0.009 |
| No metastasis | 52 | 32 | 20 |  |
| Metastasis | 36 | 12 | 24 |  |
| CEA level |  |  |  | 0.831 |
| < 5 (ng/mL) | 47 | 24 | 23 |  |
| ≥ 5 (ng/mL) | 41 | 20 | 21 |  |
| Abbreviations: CRC, colorectal cancer; CEA, carcino-embryonic antigen; * Chi-square test. | | | | |

Table S2.

| **Univariate and multivariate Cox regression analysis of different prognostic variables in CRC patients** | | | |
| --- | --- | --- | --- |
| Variable | Subset | Hazard ratio (95% CI) | *P* |
| Univariate analysis (*n* = 88) | | | |
| Age (Year) | < 60 *vs*. ≥ 60 | 1.375 (0.713-2.650) | 0.342 |
| Gender | Male *vs*. Female | 1.659 (0.815-3.377) | 0.163 |
| Tumor location | Rectum *vs*. Colon | 0.520 (0.249-1.087) | 0.082 |
| Histological grade (WHO) | G1-2 *vs*. G3 | 0.786(0.343-1.797) | 0.568 |
| Clinical stage | Ⅰ+Ⅱ *vs*. Ⅲ+Ⅳ | 2.192 (1.133-4.244) | 0.020 |
| pT status | T1-2 *vs*. T3-4 | 2.694 (1.208-6.008) | 0.015 |
| pN status | N0 *vs*. N1 | 2.192 (1.133-4.244) | 0.020 |
| CEA level | < 5 (ng/mL) *vs*. ≥ 5 (ng/mL) | 1.606 (0.833-3.098) | 0.157 |
| MEX3A expression level | Low *vs*. High | 3.553 (1.730-7.301) | 0.001 |
| Multivariate analysis (*n* = 88) | | | |
| Histological grade (WHO) | G1-2 *vs*. G3 |  | 0.400 |
| Clinical stage | Ⅰ+Ⅱ *vs*. Ⅲ+Ⅳ |  | 0.112 |
| pT status | T1-2 *vs*. T3-4 | 2.388 (1.009-5.649) | 0.048 |
| pN status | N0 *vs*. N1 |  | 0.400 |
| MEX3A expression level | Low *vs*. High | 2.462 (1.121-5.408) | 0.025 |
| Abbreviations: CRC, colorectal cancer; CEA, carcino-embryonic antigen. | | | |

Table S3.

**List of candidates MEX3A-interacting proteins identified by MS**

| Gene Name | Unique Peptides | | Coverage (%) | | Molecular Weight (kDa) | |
| --- | --- | --- | --- | --- | --- | --- |
| \| *H4C1* \| \| --- \| \| *RPL26L1* \| \| *RPS12* \| \| *DDX5* \| \| *FAM98A* \| \| *SLC25A5* \| \| ***MEX3A*** \| \| *RPS16* \| \| *RPS20* \| \| *RPS13* \| \| *RPS3* \| \| *RPS2* \| \| *RPS4X* \| \| *RPL10* \| \| *CAPRIN1* \| \| *PGAM5* \| \| *RPS8* \| \| *RPS3A* \| \| *HNRNPK* \| \| *YBX1* \| \| ***MOV10*** \| \| *H1-2* \| \| *HSPA1A* \| \| *RPL13* \| \| *RPS18* \| \| *RPS19* \| \| *PABPC4* \| \| *RPS11* \| \| *RBM14* \| \| *RTRAF* \| \| *RPL23A* \| \| *RPS14* \| \| *KHDRBS1* \| \| *TUBB* \| \| *FUS* \| \| *HNRNPH1* \| \| *ILF3* \| \| *DHX9* \| \| *DHX30* \| \| *RPS9* \| \| *RBMX* \| \| *RTCB* \| \| *DDX3X* \| \| *NRNPA2B1* \| \| *DDX17* \| \| ***PABPC1*** \| \| *IGF2BP1* \| \| *HNRNPH3* \| \| *FXR1* \| \| *RPS6* \| \| ***UPF1*** \| \| *DDX1* \| \| *NUFIP2* \| \| *DDX6* \| \| *ATXN2L* \| \| *HSPA8* \| \| *MATR3* \| \| *ATAD3B* \| \| *LARP1* \| \| *HNRNPC* \| \| *FXR2* \| \| *RPL8* \| \| *EWSR1* \| | \| 3 \| \| --- \| \| 4 \| \| 3 \| \| 3 \| \| 3 \| \| 6 \| \| 22 \| \| 6 \| \| 3 \| \| 6 \| \| 7 \| \| 5 \| \| 7 \| \| 6 \| \| 3 \| \| 5 \| \| 4 \| \| 4 \| \| 4 \| \| 5 \| \| 4 \| \| 4 \| \| 4 \| \| 4 \| \| 6 \| \| 4 \| \| 8 \| \| 7 \| \| 3 \| \| 4 \| \| 3 \| \| 3 \| \| 3 \| \| 3 \| \| 5 \| \| 4 \| \| 3 \| \| 3 \| \| 4 \| \| 6 \| \| 5 \| \| 11 \| \| 10 \| \| 4 \| \| 8 \| \| 15 \| \| 9 \| \| 6 \| \| 3 \| \| 3 \| \| 5 \| \| 16 \| \| 4 \| \| 3 \| \| 7 \| \| 3 \| \| 4 \| \| 5 \| \| 3 \| \| 3 \| \| 3 \| \| 3 \| \| 3 \| | | \| 29.1 \| \| --- \| \| 26.9 \| \| 20.5 \| \| 8.5 \| \| 7.3 \| \| 14.8 \| \| 45.4 \| \| 36.3 \| \| 22.7 \| \| 32.5 \| \| 33.3 \| \| 17.1 \| \| 19.4 \| \| 15.9 \| \| 4.1 \| \| 18 \| \| 15.4 \| \| 13.6 \| \| 9.5 \| \| 39.5 \| \| 4.1 \| \| 17.4 \| \| 12.6 \| \| 18.5 \| \| 32.9 \| \| 18.6 \| \| 19.9 \| \| 36.7 \| \| 5.4 \| \| 12.7 \| \| 21.2 \| \| 22.5 \| \| 8.4 \| \| 7.4 \| \| 12.2 \| \| 15.8 \| \| 3.4 \| \| 2.4 \| \| 3.6 \| \| 26.8 \| \| 11.3 \| \| 23.2 \| \| 19 \| \| 12.5 \| \| 14.3 \| \| 28.9 \| \| 17.9 \| \| 17.1 \| \| 6.1 \| \| 9.6 \| \| 5 \| \| 20.3 \| \| 8.6 \| \| 8.9 \| \| 8.3 \| \| 8.7 \| \| 3.2 \| \| 7.1 \| \| 3.6 \| \| 9.8 \| \| 6.5 \| \| 10.1 \| \| 6.7 \| | | 11.367  17.256  14.515  69.147  55.272  32.852  54.173  16.445  13.373  17.222  26.688  31.324  29.597  24.604  78.365  32.004  24.205  29.945  50.976  35.924  113.67  21.364  70.051  24.261  17.718  16.06  70.782  18.431  69.491  28.068  17.695  16.273  48.227  49.67  53.425  49.229  95.337  140.96  133.94  22.591  42.331  55.21  73.243  37.429  80.272  70.67  63.48  36.926  69.72  28.68  124.34  82.431  76.12  54.416  113.37  70.897  94.622  72.572  123.51  33.67  74.222  28.024  68.477 | |
|  | |  | |  | |  |

Table S4.

**List of primers, and shRNA sequences**

| Name | Primer sequence (5’-3’) |  |
| --- | --- | --- |
| MEX3A-Forward  MEX3A-Reverse  circMPP6 convergent-Forward | CAAGCTCTGCGCTCTCTACAAA  GGCCTTAATCTTGCAGCCTTG  GAGCTGCCCTCGTCTACTG |  |
| circMPP6 convergent-Reverse | CTGGAAGGGAGGCATTTTGG |  |
| circMPP6 divergent-Forward | CCAAAATGCCTCCCTTCCAG |  |
| circMPP6 divergent-Reverse | CAGTAGACGAGGGCAGCTC |  |
| PDE5A-Forward | TGAACATCCACTTGCCCAGC |  |
| PDE5A-Reverse | GAGAATCTGATTGCCTGGACT |  |
| SENP7-Forward | TCGTCTCACTGGTATCTCGC |  |
| SENP7-Reverse | CATTTTGGGACTGCTGAGCCC |  |
| TTLL7-Forward | CAAATGTTGCCGGGACAAAG |  |
| TTLL7-Reverse  GEN1-Forward  GEN1-Reverse  ASXL2-Forward  ASXL2-Reverse  BIRC2-Forward  BIRC2-Reverse  BRCC3-Forward  BRCC3-Reverse  MTAP-Forward  MTAP-Reverse  SMN2-Forward  SMN2-Reverse  ZNF664-Forward  ZNF664-Reverse | TCCTTCCTACAGATCTCCCC  TCCCCTTGCGTAATCTTGGTG  TCCAGAAGACCCATACCGAGA  GGAAAAGGGACGTAGGAAGAAG  ACTCATGGGTGTATTGGGGTA  ACGAATGAAAGGCCAAGAGT  GGTGGGTCAGCATTTTCTTCT  GAGTCTGACGCTTTCCTCGTT  TGTATCATCGTTCAACTCCCCT  ACCACCGCCGTGAAGATTG  GCATCAGATGGCTTGCCAA  GCCCAAATCTGCTCCATGG  TAGTAAGTGGGGTGGTGGTG  TCTACAAGTGCCCCATGTGT  GCTCCAATTGAAGGCTTTGC |  |
| GAPDH-Forward | TGCACCACCAACTGCTTAGC |  |
| GAPDH-Reverse | GGCATGGACTGTGGTCATGAG |  |
| U3-Forward | TTCTCTGAGCGTGTAGAGCACCGA |  |
| U3-Reverse | GATCATCAATGGCTGACGGCAGTT |  |

| Name | Sequence (5’-3’) |
| --- | --- |
| sh-MEX3A-1 | CCTGTCGAGAATTAACACCTC |
| sh-MEX3A-2 | GCTAGTTTAAGATTGGGAGCT |
| sh-circMPP6 | AACTACGGTGCCATCAATGCA |
| sh-PDE5A | CCAGCTTTACTGCCATTCAAT |

Movie S1.

FRAP of EGFP-MEX3A-FL droplet formed in DLD-1 cells. An EGFP-MEX3A-FL droplet was bleached. Recovery was recorded for every 0.5 s after bleaching.

Movie S2.

FRAP of EGFP-MEX3A-FL droplet formed in SW480 cells. An EGFP-MEX3A-FL droplet was bleached. Recovery was recorded for every 0.5 s after bleaching.

Movie S3.

FRAP of EGFP-MEX3A-IDRs droplet formed in DLD-1 cells. An EGFP-MEX3A-IDRs droplet was bleached. Recovery was recorded for every 0.5 s after bleaching.

Movie S4.

FRAP of EGFP-MEX3A-IDRs droplet formed in SW480 cells. An EGFP-MEX3A-IDRs droplet was bleached. Recovery was recorded for every 0.5 s after bleaching.

Movie S5.

FRAP of EGFP-MEX3A-IDRs△ punctum formed in DLD-1 cells. An EGFP-MEX3A-IDRs△ punctum was bleached. Recovery was recorded for every 0.5 s after bleaching.

Movie S6.

FRAP of EGFP-MEX3A-IDRs△ punctum formed in SW480 cells. An EGFP-MEX3A-IDRs△ punctum was bleached. Recovery was recorded for every 0.5 s after bleaching.

Other Supplementary Materials for this manuscript include the following:

Original and uncropped films of Western blotting


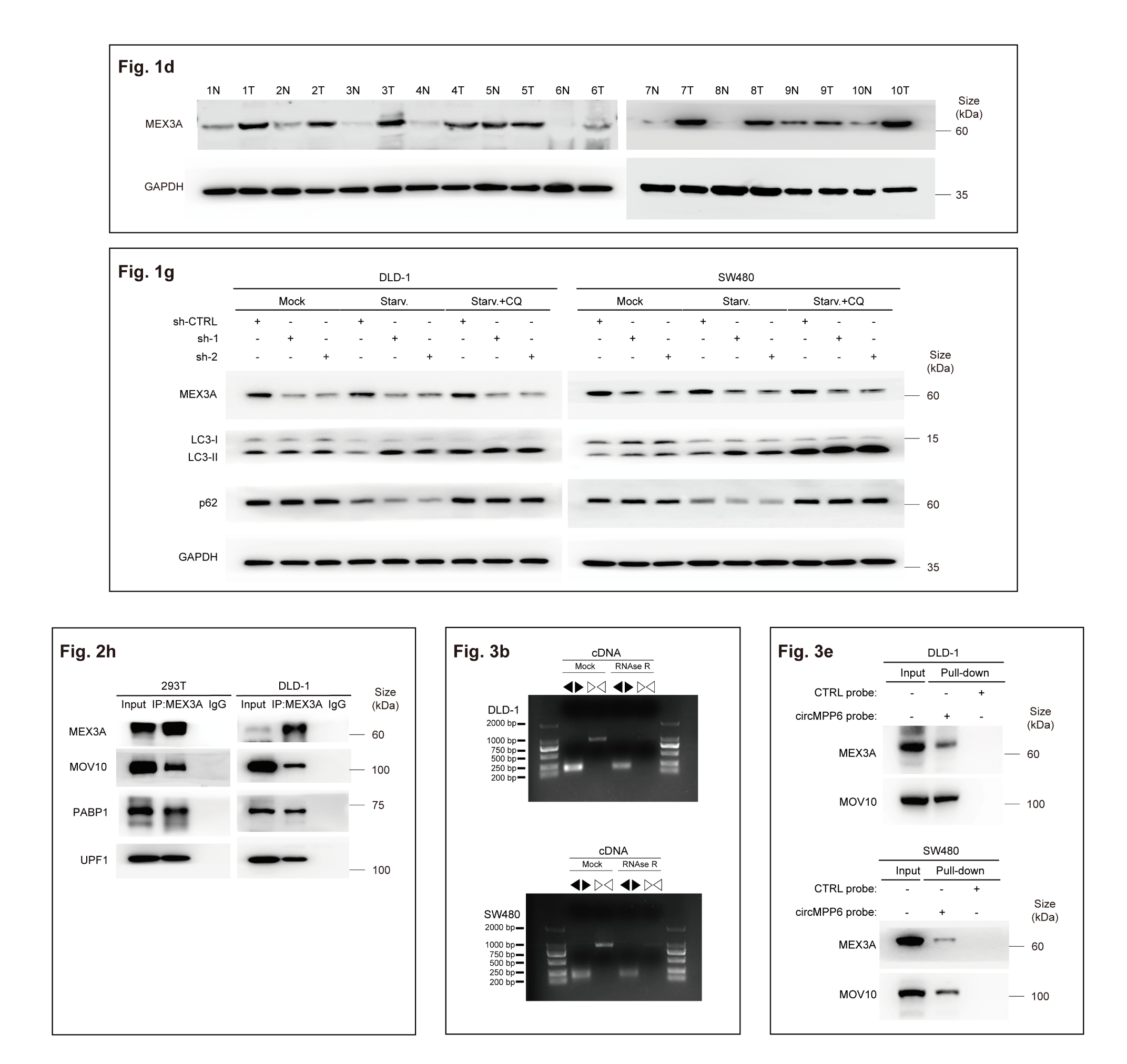


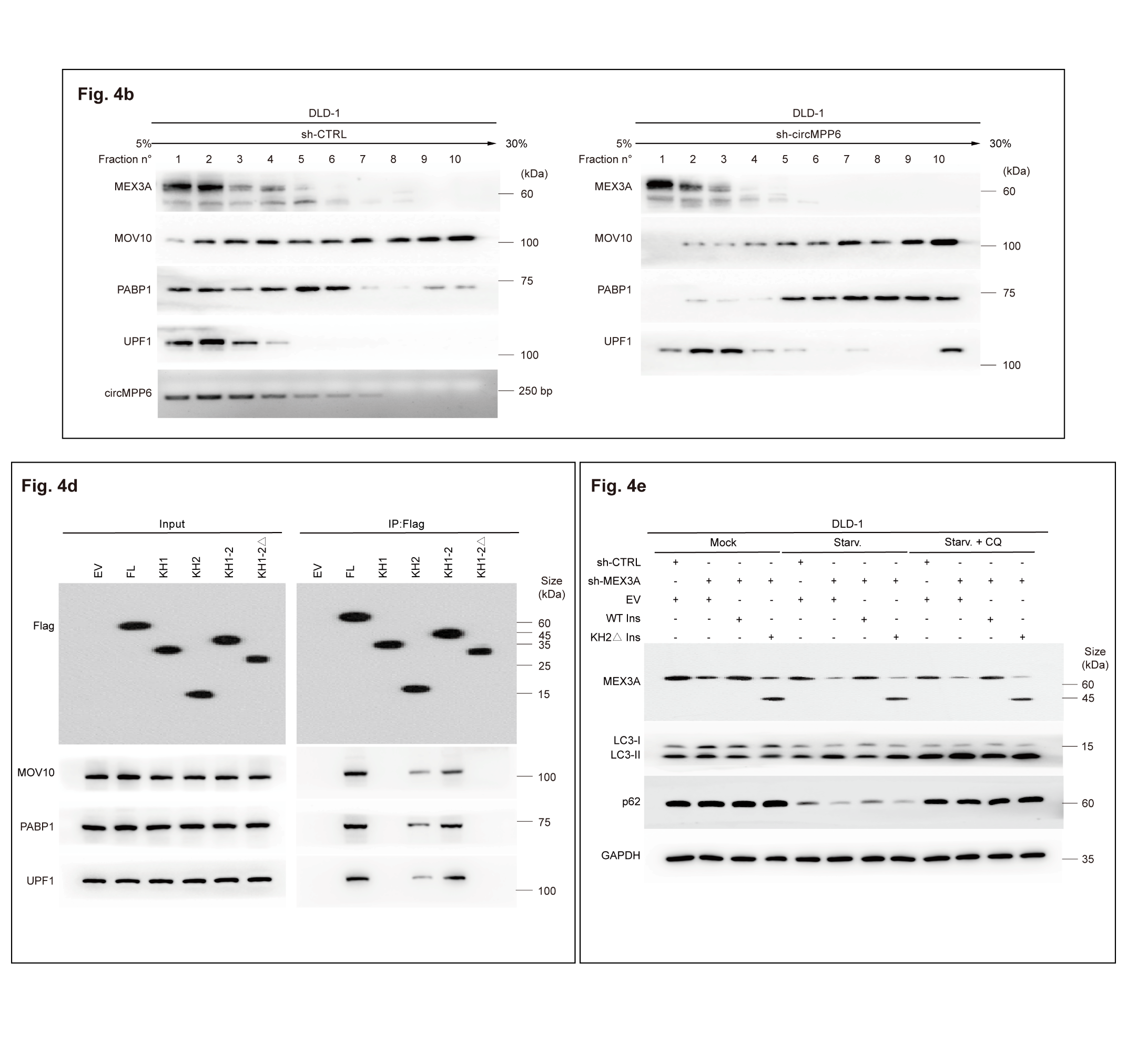


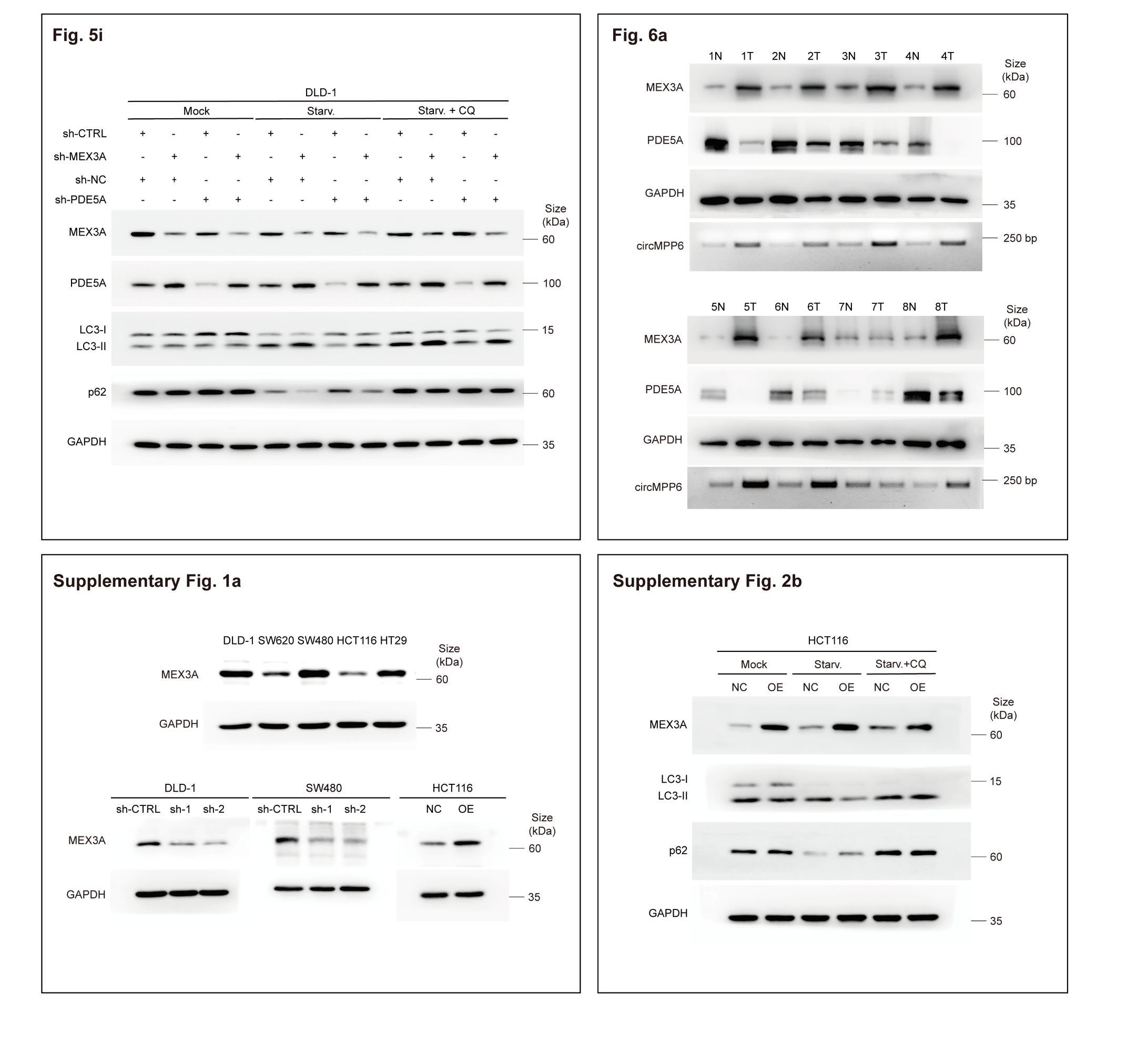


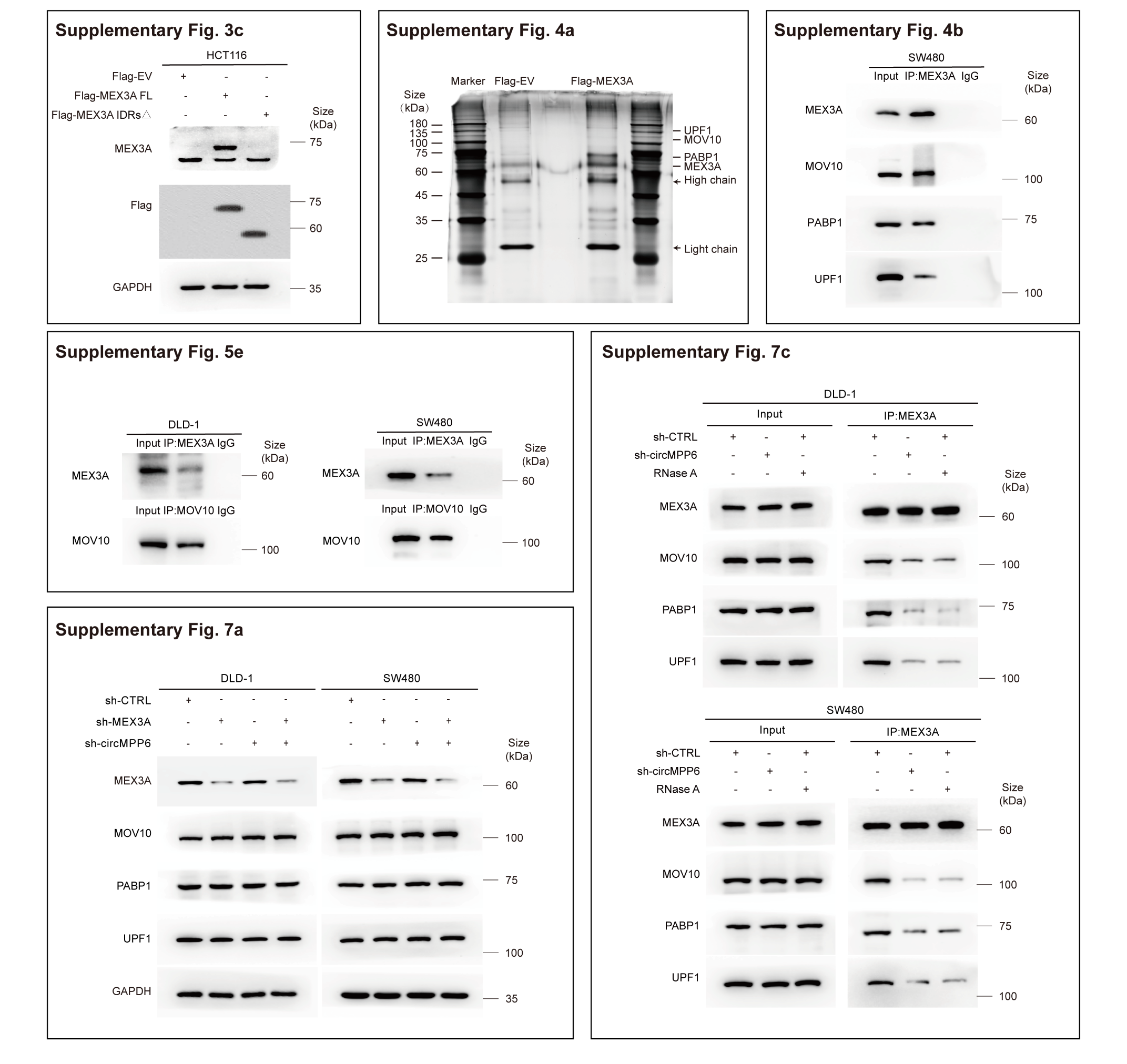


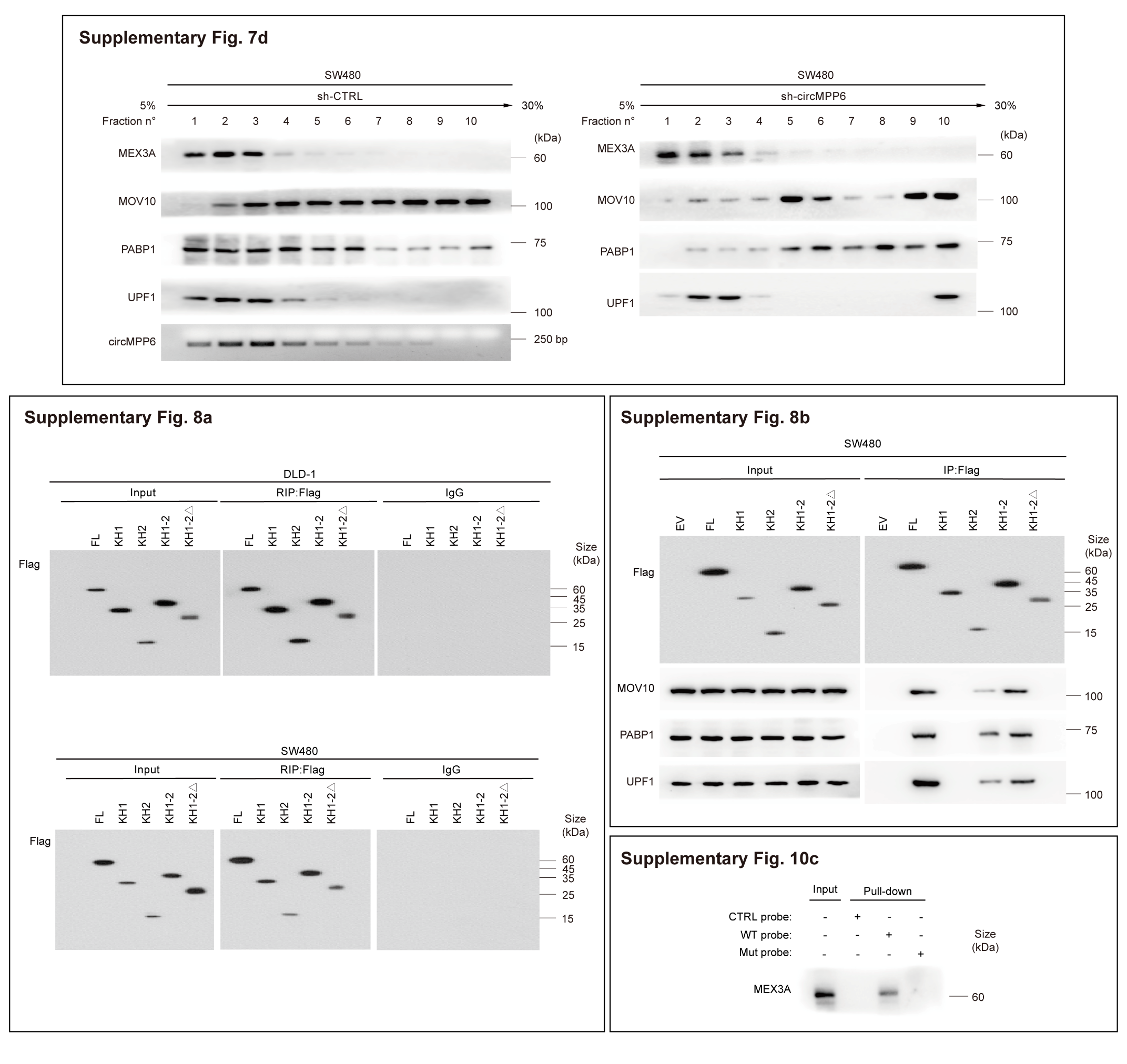


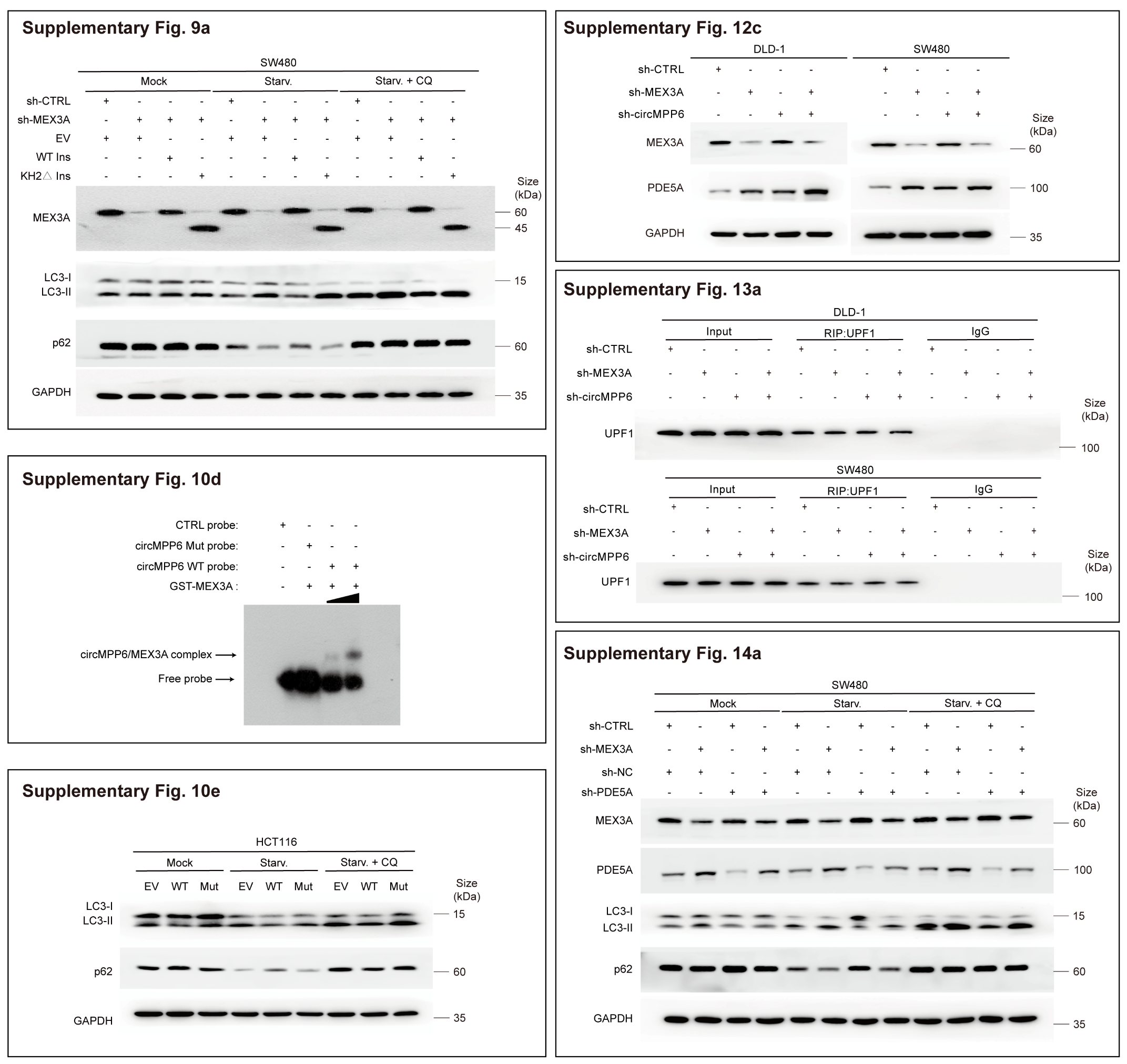


Original Microscopy images.


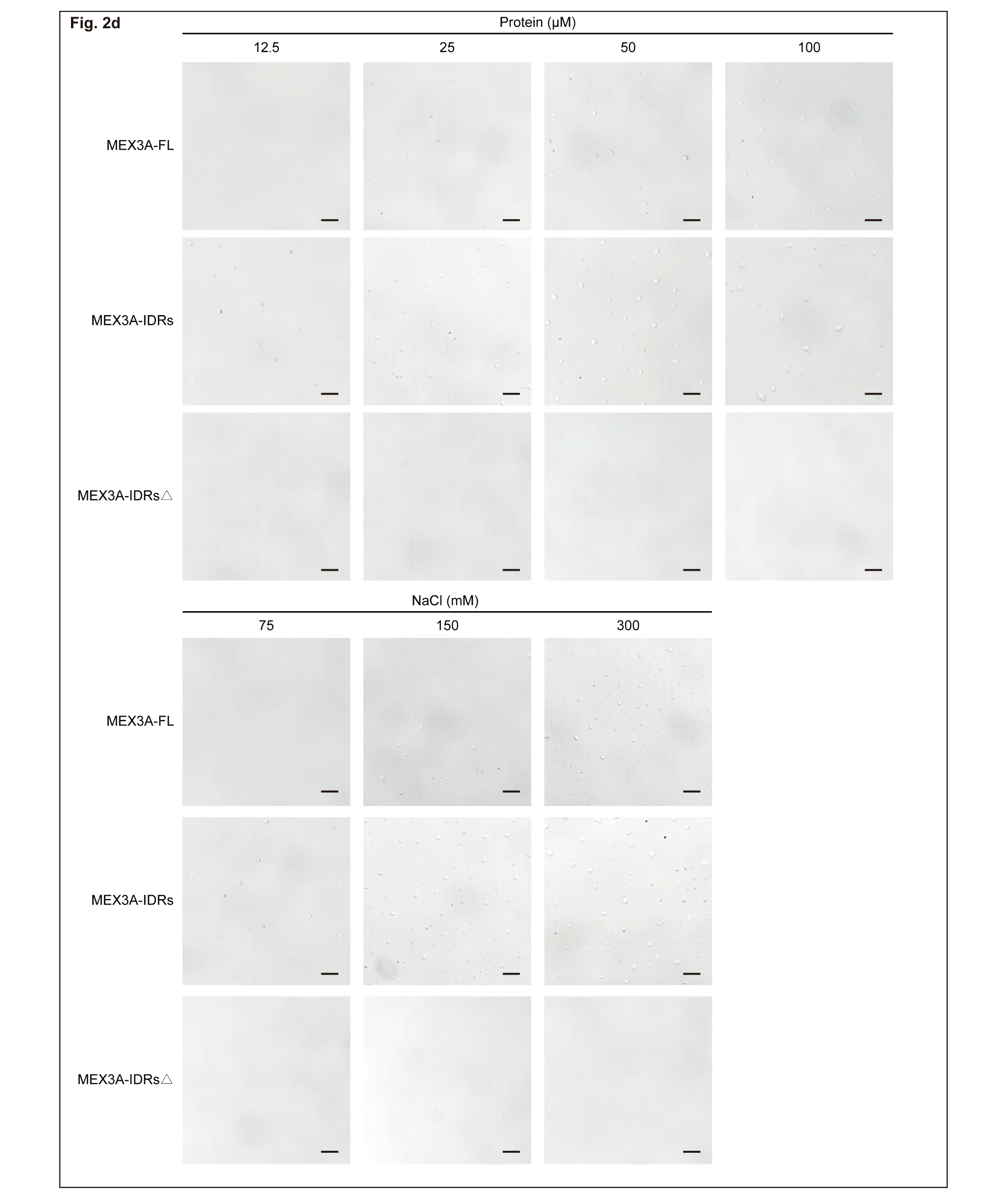


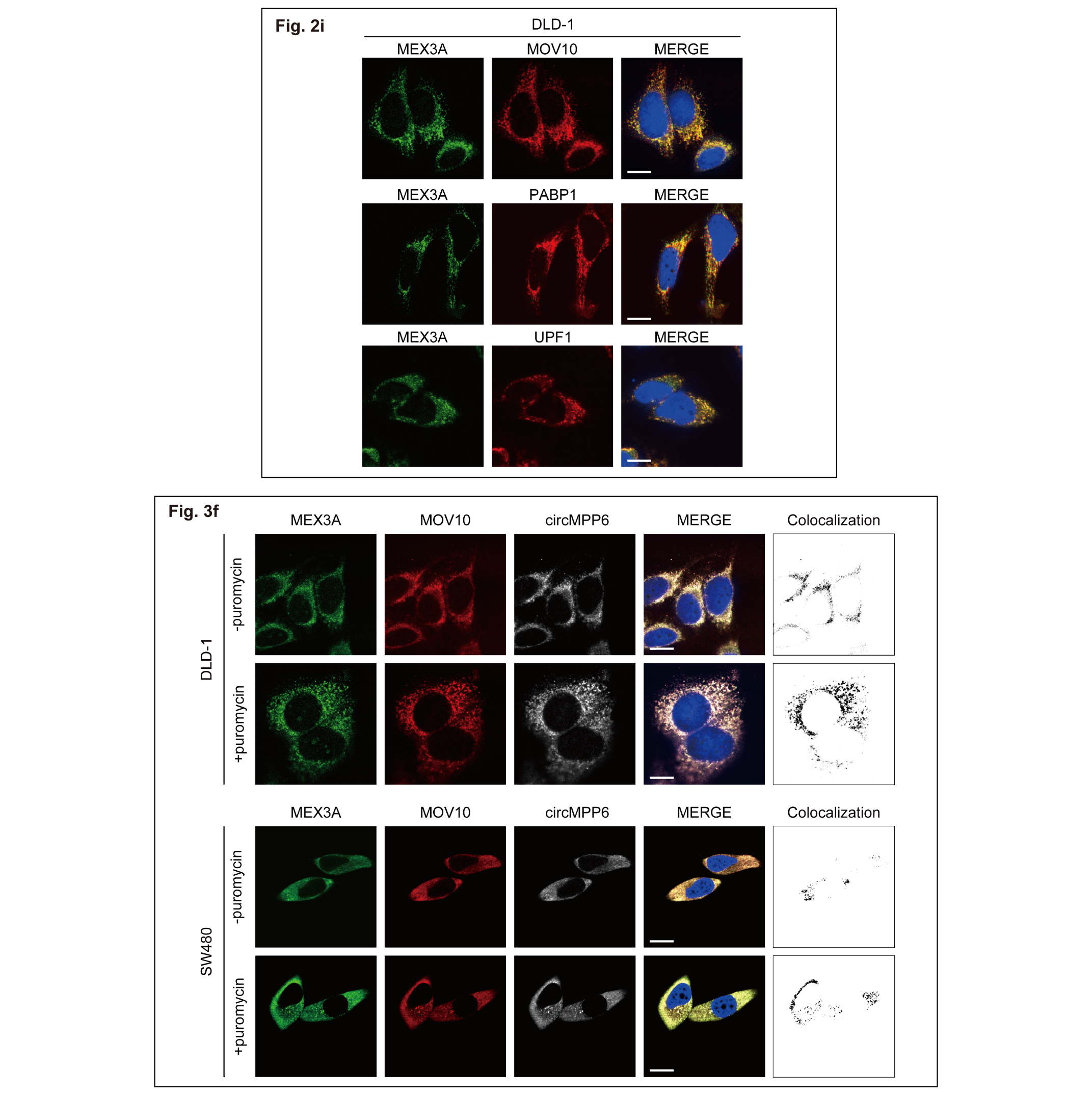


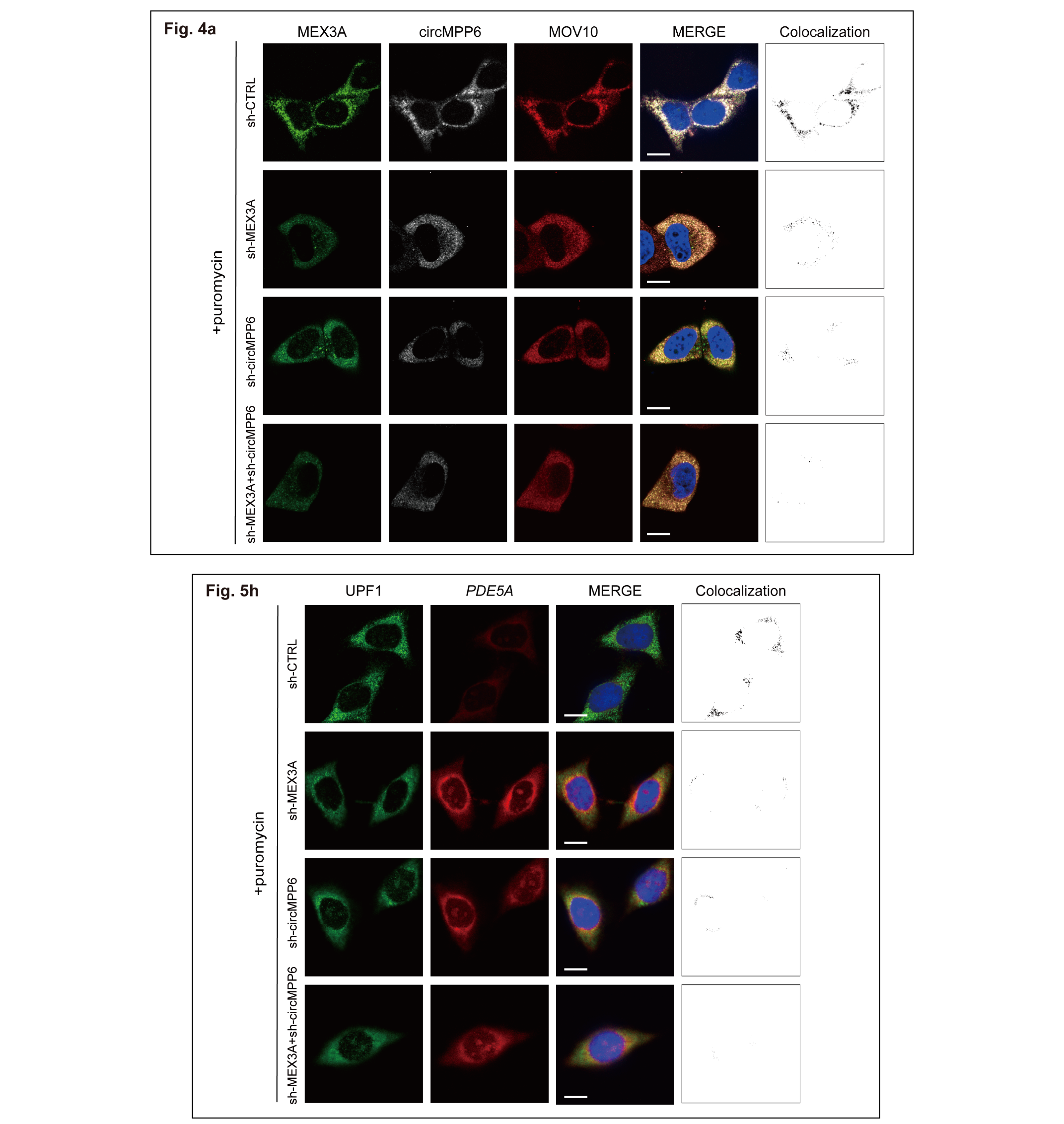


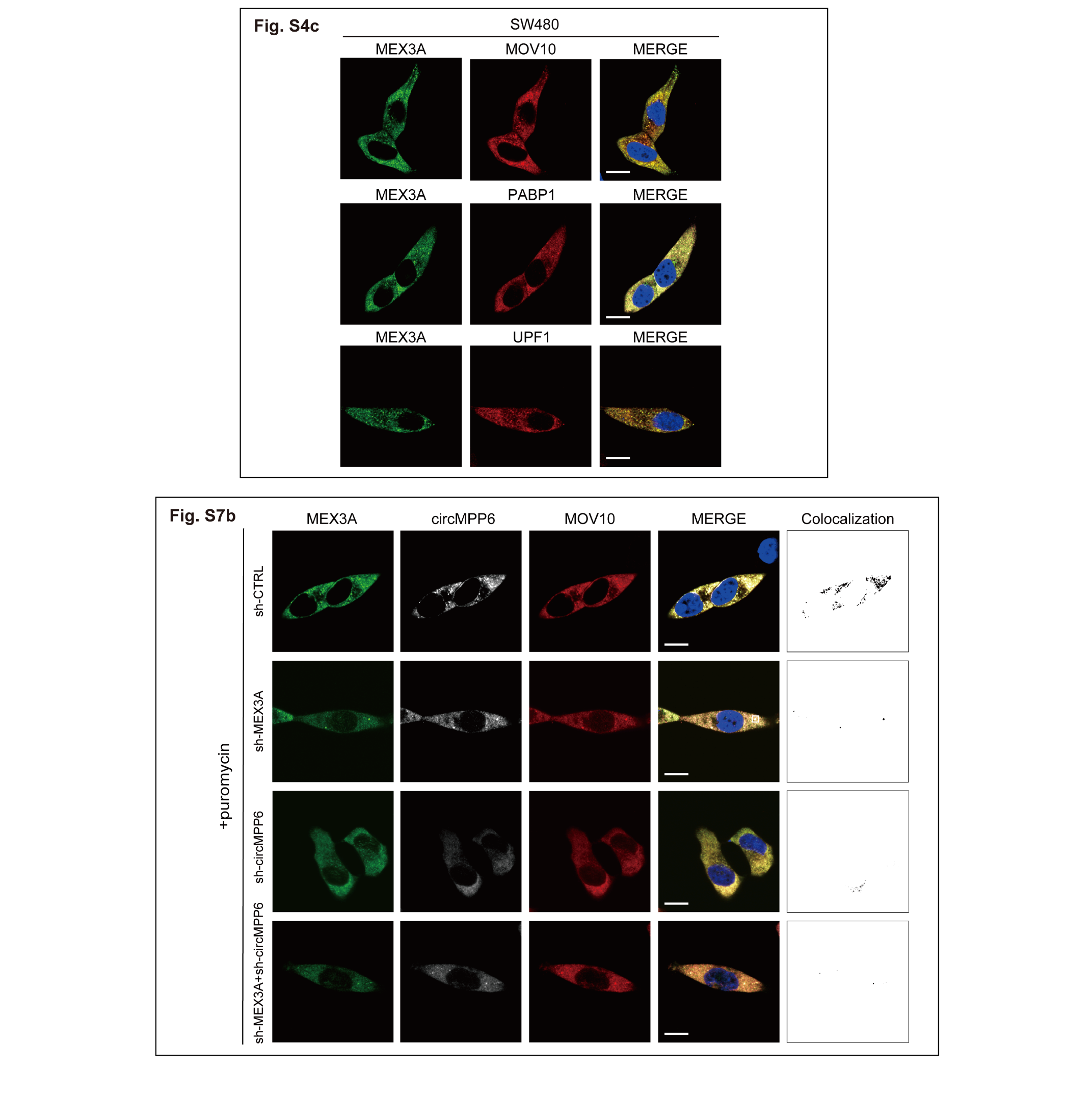


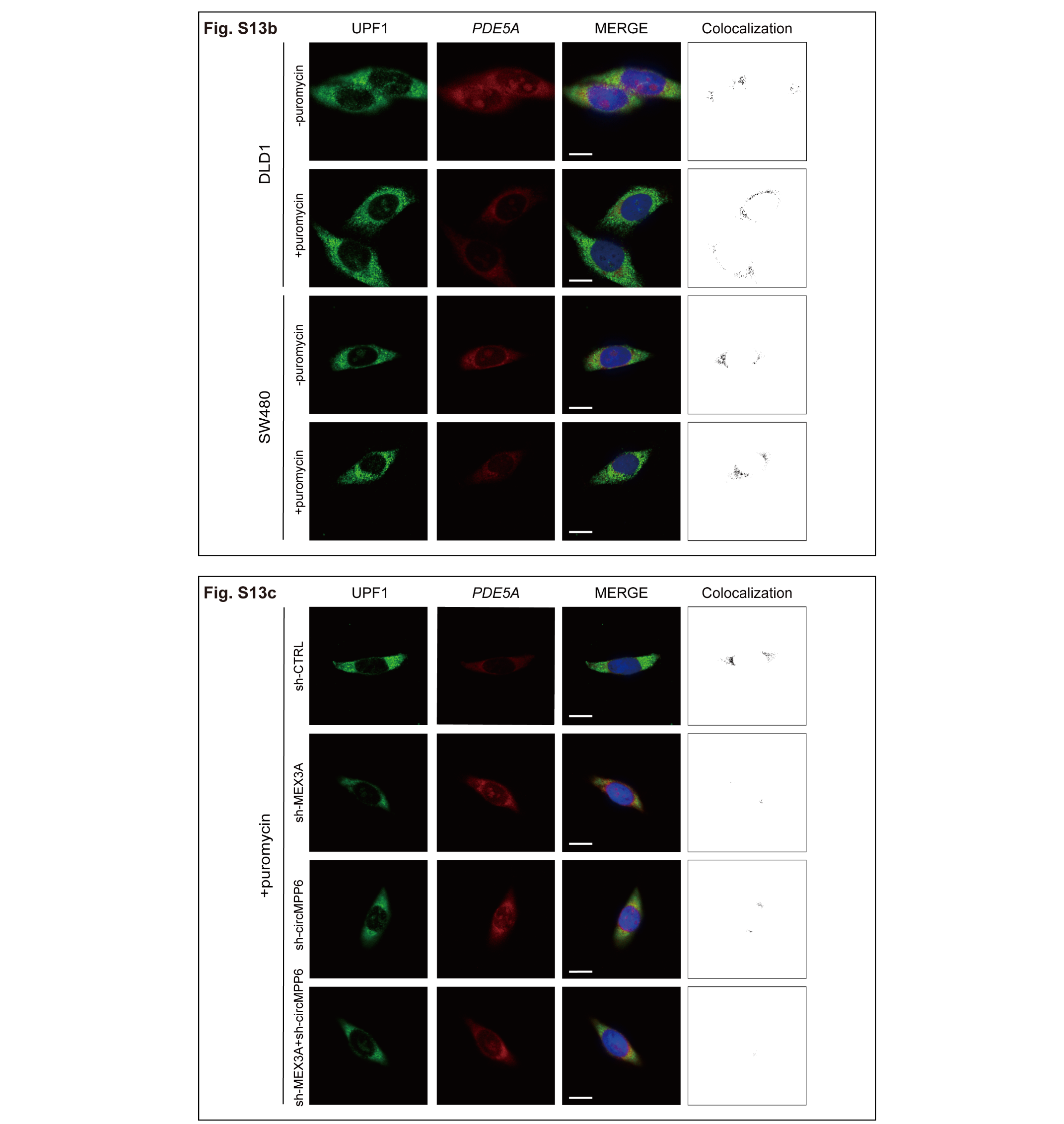

Supplement: Supplementary file 1 — Supplementary_Materials [file 41392_2024_1787_MOESM1_ESM.docx]
